# Supplementary material for: Functional characterization and structural bases of two class I diterpene synthases in pimarane-type diterpene biosynthesis
Source: Commun Chem. 2021 Sep 30;4:140. doi: 10.1038/s42004-021-00578-z (PMC9814573; doi:10.1038/s42004-021-00578-z)
Supplement: Supplementary file 2 — Supplementary Information [file 42004_2021_578_MOESM2_ESM.pdf]

Supplementary Information

Functional Characterization and Structural Bases of Two Class I Diterpene Synthases in Pimarane-Type Diterpenes Biosynthesis

Baiying Xing[1,#], Jiahui Yu[1,#], Changbiao Chi[1], Xueyang Ma[1], Qingxia Xu[1], Annan Li[1], Yuanjie Ge[1], Zhengdong Wang[1], Tan Liu[1], Hongli Jia[1], Fuling Yin[1], Juan Guo[2], Luqi Huang[2], Donghui Yang[1,*], and Ming Ma [1,*]

[1]State Key Laboratory of Natural and Biomimetic Drugs, School of Pharmaceutical Sciences, Peking University, 38 Xueyuan Road, Haidian District, Beijing 100191, China

[2]State Key Laboratory of Dao-di Herbs, National Resource Center for Chinese Materia Medica, China Academy of Chinese Medical Sciences, Beijing 100700, China

[#]These authors contributed equally

*To whom correspondence should be addressed: Donghui Yang, Email: ydhui@bjmu.edu.cn; Ming Ma, Email: mma@bjmu.edu.cn

Table of Contents

Table S1. The functional annotation of the *sat* and *stt* biosynthetic genes ······S3
Table S2. Primers for plasmids construction in this study ······S4
Table S3. Plasmids and strains used in this study ······S6
Table S4. The information of synthesized genes in the construction of engineered *E. coli* strains ····S9
Table S5. The ^{1}H NMR data of **1-9** in $CDCl_3$ ······S10
Table S6. The ^{13}C NMR data of **1-9** in $CDCl_3$ ······S12
Table S7. The data collection and refinement statistics of crystal structures ······S13
Figure S1. Representative pimarane-type diterpenoids with different absolute configurations ······S14
Figure S2. The reported structures of DTSs and their catalytic reactions ······S15
Figure S3. The degenerate primers designed for genome mining ······S17
Figure S4. The phylogenetic analysis of strains PKU-MA00418 and PKU-TA00600 ······S18
Figure S5. Comparison between *sat* gene cluster and *terp1* gene cluster ······S19
Figure S6. The construction of engineered *E. coli* strains ······S21
Figure S7. Mass spectra of compounds **1-9** identified from GC-MS analysis ······S22
Figure S8. The SDS-PAGE of Sat1646 and Stt4548 and the TLC analysis of compound **2** ······S23
Figure S9. The crystallographic packing of Sat1646-Mg^{2+} ······S24
Figure S10. The comparison between Sat1646-Mg^{2+} and apo-Sat1646 in general structures, motifs and B factor values ······S25
Figure S11. The crystallographic packing of Stt4548 and comparison with Sat1646-Mg^{2+} ······S26
Figure S12. GC-MS analysis of the products of Sat1646 mutants with SmCPS ······S27
Figure S13. GC-MS analysis of the products of Stt4548 mutants with SmCPS ······S28
Figure S14. The proposed biosynthetic pathways of **2-7** ······S29
Figure S15. The ^{1}H NMR and ^{13}C NMR spectrum of compound **1** in $CDCl_3$ ······S30
Figure S16. The ^{1}H NMR and ^{13}C NMR spectrum of compound **2** in $CDCl_3$ ······S31
Figure S17. The COSY spectrum of compound **2** in $CDCl_3$ ······S32
Figure S18. The HSQC spectrum of compound **2** in $CDCl_3$ ······S32
Figure S19. The HMBC spectrum of compound **2** in $CDCl_3$ ······S33
Figure S20. The NOESY spectrum of compound **2** in $CDCl_3$ ······S33
Figure S21. The DEPT 135° spectrum of compound **2** in $CDCl_3$ ······S34
Figure S22. The IR spectrum of compound **2** ······S34
Figure S23. The HREIMS spectrum of compound **2** ······S35
Figure S24. The ^{1}H NMR and ^{13}C NMR spectrum of compound **3** in $CDCl_3$ ······S36
Figure S25. The ^{1}H NMR and ^{13}C NMR spectrum of compound **4** in $CDCl_3$ ······S37
Figure S26. The ^{1}H NMR and ^{13}C NMR spectrum of compound **5** in $CDCl_3$ ······S38
Figure S27. The ^{1}H NMR and ^{13}C NMR spectrum of compound **6** in $CDCl_3$ ······S39
Figure S28. The ^{1}H NMR and ^{13}C NMR spectrum of compound **7** in $CDCl_3$ ······S40
Figure S29. The ^{1}H NMR and ^{13}C NMR spectrum of compound **8** in $CDCl_3$ ······S41
Figure S30. The ^{1}H NMR and ^{13}C NMR spectrum of compound **9** in $CDCl_3$ ······S42
Supplementary References ······S43

Table S1. The functional annotation of the *sat* and *stt* biosynthetic genes.

Genes	Annotation based on BLAST			
	Numbers of amino acids	Proposed functions	Accession numbers of closest homologues	Identities with homologues
		sat gene cluster		
sat1645	531	copalyl diphosphate synthase	WP_012181499.1	98.12%
sat1646	295	pimaradiene synthase	WP_012181498.1	98.64%
sat1647	448	cytochrome P450	WP_018798930.1	98.44%
		stt gene cluster		
stt4536	385	MULTISPECIES: acetyl-CoA acetyltransferase	WP_056560797.1	91.69%
stt4537	262	SDR family oxidoreductase	WP_067270392.1	90.04%
stt4538	212	TetR family transcriptional regulator	WP_019885445.1	92.12%
stt4539	381	acyl-CoA dehydrogenase	WP_017947508.1	89.82%
stt4540	327	acyl-CoA dehydrogenase	WP_055616992.1	68.28%
stt4541	156	Pyridoxamine 5'-phosphate oxidase	WP_138055895.1	78.21%
stt4542	493	copalyl diphosphate synthase	AHK61132.1	44.12%
stt4543	338	geranylgeranyl diphosphate synthase, type I	SCE38651.1	53.71%
stt4544	63	MULTISPECIES: protein phosphatase	WP_070011137.1	36.07%
stt4545	613	squalene-hopene cyclase	WP_057236678.1	50.16%
stt4546	251	SDR family oxidoreductase	WP_051861443.1	50.00%
stt4547	198	acetoacetate decarboxylase (ADC)	SEQ15092.1	45.56%
stt4548	292	pimaradiene synthase	AHK61133.1	30.00%
stt4549	125	glyoxalase/bleomycin resistance/dioxygenase family protein	WP_031138160.1	76.00%
stt4550	240	toxic cation resistance protein	WP_019885454.1	89.03%

Table S2. Primers for plasmids construction in this study.

Primers	Sequence (5' to 3')
idi-pRSFDuet-F	GAGATATACCATGGATGCAAACGGAACACGTC
idi-pRSFDuet-R	CTCGAATTCGGATCCTTATTTAAGCTGGGTAAATGCAG
dxr-pRSFDuet-F	GAAGGAGATATACATATGAAGCAACTCACCATTCTGG
dxr-pRSFDuet-R	CAGACTCGAGGGTACCTCAGCTTGCGAGACGCATC
dxs-pRSFDuet-F	CTGAGGTACCCTCGAGATGAGTTTTGATATTGCCAAATAC
dxs-pRSFDuet-R	GCCTAGGTTAATTAATTATGCCAGCCAGGCCTTG
pRSFDuet-idi-F	GGATCCGAATTCGAGCTC
pRSFDuet-idi-R	CCATGGTATATCTCCTTATTAAAGTTAAAC
pRSFDuet-dxr-F	GGTACCCTCGAGTCTGGTAAAG
pRSFDuet-dxr-R	CATATGTATATCTCCTTCTTATACTTAACTAATATAC
pRSFDuet-dxs-F	TTAATTAACCTAGGCTGCTGCCACCGC
pRSFDuet-dxs-R	CTCGAGGGTACCTCAGCTTGCGAG
PtmT4-pCDFDuet-F	GGAGATATACATATGGTTCACGCTGACACCGTTCAG
PtmT4-pCDFDuet-R	CTTTACCAGACTCGAGTTAGTGTTTACGGAAAGCAACGTAGTC
pCDFDuet-PtmT4-F	CTCGAGTCTGGTAAAGAAACCGCTG
pCDFDuet-PtmT4-R	CATATGTATATCTCCTTCTTATACTTAACTAATATACTAAGATGGGG
Sat1646-pCDFDuet-F	ATAAGGAGATATACCATGGTGCCAACACACCACGCCGG
Sat1646-pCDFDuet-R	GCGGCCGCAAGCTTTTAACGTTCAGAGTCACCCCAGTAGTCCGC
PCDFDuet-Sat1646-F	AAGCTTGCGGCCGCATAATG
PCDFDuet-Sat1646-R	CATGGTATATCTCCTTATTAAAGTTAAACAAAATTATTTCTAC
1645-pCDFDuet-F	AAGCTTGCGGCCGCGTGTCTGCTGACCTGGGTGC
1645-pCDFDuet-R	CGACTTAAGCATTATTTAAGCGGTAGCCGGACCAGC
pCDFDuet-1645-F	ATAATGCTTAAGTCGAACAGAAAGTAATCGTATTG
pCDFDuet-1645-R	GCGGCCGCAAGCTTTTAACGTTC
SmCPS-pCDFDuet-F	AAGCTTGCGGCCGCATGGCCTCCTTATCCTCTACAATCC
SmCPS-pCDFDuet-R	CGACTTAAGCATTATTCACGCGACTGGCTCGAAAAGC
OsCPS4-pCDFDuet-F	AAGCTTGCGGCCGCATGCTGATCTCTAAATCTCCGC
OsCPS4-pCDFDuet-R	CGACTTAAGCATTATTTAGATAACGTCCTGGAAGATAACTTTG
Stt4548-pCDFDuet-F	ATAAGGAGATATACCATGATGACCACGGACAATGCCGATCAG
Stt4548-pCDFDuet-R	GCGGCCGCAAGCTTTCATCCGGCGGTATCGAGGAGCG
4542-pCDFDuet-F	AAGCTTGCGGCCGCATGGACACCGCTGACGACCT
4542-pCDFDuet-R	CGACTTAAGCATTATTTACTGAGCACGTTCAGCTTCTTCAGC
PtmT2-pCDFDuet-F	AAGCTTGCGGCCGCATGCTGGAAGTTCCGGCTCAGC
PtmT2-pCDFDuet-R	CGACTTAAGCATTATTTAAGCACCACCAGAAGCAGCAGAAG
Haur_2145-pCDFDuet-F	AAGCTTGCGGCCGCATGTCTCTGATCGTTGACATCCTG
Haur_2145-pCDFDuet-R	CGACTTAAGCATTATTTACAGTTTCAGTTCACCCAGGG
Sat1646-L58A-F	ACGATGCTGTCTGCTGCTGCTCTGTCTACC
Sat1646-L58A-R	AGCAGCAGACAGCATCGTGTCATCGACCGG
Sat1646-S81A-F	GTTGCTAACCGTGCTGCTCTGTGGGTTACC
Sat1646-S81A-R	AGCAGCACGGTTAGCAACACGCAGCTGTTC
Sat1646-V84G-F	CGTGCTTCTCTGTGGGGTACCGCTGAAGAC

Sat1646-V84G-R	ACCCCACAGAGAAGCACGGTTAGCAACACG
Sat1646-T85A-F	GCTTCTCTGTGGGTTGCTGCTGAAGACTGG
Sat1646-T85A-R	AGCAACCCACAGAGAAGCACGGTTAGCAAC
Sat1646-Y183A-F	GACAACGCTGACAACGCTGGTGCTTCTTTC
Sat1646-Y183A-R	AGCGTTGTCAGCGTTGTCCAGGTATTCAGC
Sat1646-G184F-F	GTTAACGAAAGAAGCGAAGTAGTTGTCAGCGTTGTC
Sat1646-G184F-R	TTCGCTTCTTTCGTTAACGTTTCTCACTGGATCGTTACC
Sat1646-A185S-F	CGTTAACGAAAGAAGAACCGTAGTTGTCAGCG
Sat1646-A185S-R	TCTTCTTTCGTTAACGTTTCTCACTGGATCGTTACCG
Sat1646-V188A-F	CAGTGAGAAACGTTAGCGAAAGAAGCACCG
Sat1646-V188A-R	GCTAACGTTTCTCACTGGATCGTTACCGGTGAC
Sat1646-Y288A-F	TTTACCACCGGCTTCGCCCACGGTGC
Sat1646-Y288A-R	AGCGAAGCCGGTGGTAAAGCCAGCTTCAC
Sat1646-Y183F-F	ACGAAAGAAGCACCGAAGTTGTCAGC
Sat1646-Y183F-R	TTCGGTGCTTCTTTCGTTAACGTTTCTCACTGG
Sat1646-Y288F-F	TTACCACCGGCTTCTTCCACGGTGC
Sat1646-Y288F-R	AAGAAGCCGGTGGTAAAGCCAGCTTC
Stt4548-R65A-F	TCCGATGTCGCCCTGATCGGGATCGGCGTGTTCACGGC
Stt4548-R65A-R	CCCGATCAGGGCGACATCGGACACGACTCCGCCGTACC
Stt4548-C95A-F	GCCGCCTGGGCCTGCGCCCTCGACGACTGCGC
Stt4548-C95A-R	GAGGGCGCAGGCCCAGGCGGCGGTCCGTGC
Stt4548-C96A-F	GCCTGGTGCGCCGCCCTCGACGACTGCGCCGAC
Stt4548-C96A-R	GTCGAGGGCGGCGCACCAGGCGGCGGTCCGTG
Stt4548-C95A/C96A-F	GACCGCCGCCTGGGCAGCCGCCCTCGACGACTG
Stt4548-C95A/C96A-R	GGCTGCCCAGGCGGCGGTCCGTGCGGGAAG
Sat1646-pMCSG19-F	TACTTCCAATCCAATGCCATGGTGCCAACACACCACG
Sat1646-pMCSG19-R	TTATCCACTTCCAATGTTAACGTTCAGAGTCACCCCAGTAG
Stt4548-pET28a-F	GGGAATTCCATATGGAATTCCCATGACCACGGACAATGCCGATC
Stt4548-pET28a-R	CCGGAATTCCGGTCATCCGGCGGTATCGAGGAGC

Table S3. Plasmids and strains used in this study.

Name	Description	Source
**Plasmids**		
pRSFDuet-1	Gene expression plasmid used in *E. coli*, containing two multiple cloning sites; kanamycin resistance	Novagen
pCDFDuet-1	Gene expression plasmid used in *E. coli*, containing two multiple cloning sites; streptomycin resistance	Novagen
pMCSG19	Gene expression plasmid used in *E. coli*, encoding N-terminal His_6 tag, ampicillin resistance	Beijing Tiandz Biotech Co., Ltd
pET28a (+)	Gene expression plasmid used in *E. coli*, encoding N-terminal His_6 tag, kanamycin resistance	Beijing Tiandz Biotech Co., Ltd
pMM-1	pRSFDuet-1-derived plasmid bearing the *idi*, *dxr*, *dxs* gene	[7]
pMM-2	pCDFDuet-1-derived plasmid bearing the *ptmT4* gene	This study
pMM-3	pCDFDuet-1-derived plasmid bearing the *sat1645*, *sat1646*, *ptmT4* gene	This study
pMM-4	pCDFDuet-1-derived plasmid bearing the *smCPS*, *sat1646*, *ptmT4* gene	This study
pMM-5	pCDFDuet-1-derived plasmid bearing the *osCPS4*, *sat1646*, *ptmT4* gene	This study
pMM-6	pCDFDuet-1-derived plasmid bearing the *stt4542*, *stt4548*, *ptmT4* gene	This study
pMM-7	pCDFDuet-1-derived plasmid bearing the *smCPS*, *stt4548*, *ptmT4* gene	This study
pMM-8	pCDFDuet-1-derived plasmid bearing the *osCPS4*, *stt4548*, *ptmT4* gene	This study
pMM-9	pCDFDuet-1-derived plasmid bearing the *ptmT2*, *stt4548*, *ptmT4* gene	This study
pMM-10	pCDFDuet-1-derived plasmid bearing the *haur_2145*, *stt4548*, *ptmT4* gene	This study
pMM-11	pCDFDuet-1-derived plasmid bearing the *kgTPS*, *stt4548*, *ptmT4* gene	This study
pMM-12	pCDFDuet-1-derived plasmid bearing the *mtHPS*, *stt4548*, *ptmT4* gene	This study
pMM-13	pCDFDuet-1-derived plasmid bearing the *smCPS*, *sat1646-L58A*, *ptmT4* gene	This study
pMM-14	pCDFDuet-1-derived plasmid bearing the *smCPS*, *sat1646-S81A*, *ptmT4* gene	This study
pMM-15	pCDFDuet-1-derived plasmid bearing the *smCPS*, *sat1646-V84G*, *ptmT4* gene	This study
pMM-16	pCDFDuet-1-derived plasmid bearing the *smCPS*, *sat1646-T85A*, *ptmT4* gene	This study

pMM-17	pCDFDuet-1-derived plasmid bearing the *smCPS*, *sat1646-Y183A*, *ptmT4* gene	This study
pMM-18	pCDFDuet-1-derived plasmid bearing the *smCPS*, *sat1646-G184F*, *ptmT4* gene	This study
pMM-19	pCDFDuet-1-derived plasmid bearing the *smCPS*, *sat1646-A185S*, *ptmT4* gene	This study
pMM-20	pCDFDuet-1-derived plasmid bearing the *smCPS*, *sat1646-V188A*, *ptmT4* gene	This study
pMM-21	pCDFDuet-1-derived plasmid bearing the *smCPS*, *sat1646-Y288A*, *ptmT4* gene	This study
pMM-22	pCDFDuet-1-derived plasmid bearing the *smCPS*, *sat1646-Y183A/Y288A*, *ptmT4* gene	This study
pMM-23	pCDFDuet-1-derived plasmid bearing the *smCPS*, *sat1646-Y288F*, *ptmT4* gene	This study
pMM-24	pCDFDuet-1-derived plasmid bearing the *smCPS*, *sat1646-Y183F*, *ptmT4* gene	This study
pMM-25	pCDFDuet-1-derived plasmid bearing the *smCPS*, *sat1646-Y183F/Y288F*, *ptmT4* gene	This study
pMM-26	pCDFDuet-1-derived plasmid bearing the *smCPS*, *stt4548-R65A*, *ptmT4* gene	This study
pMM-27	pCDFDuet-1-derived plasmid bearing the *smCPS*, *stt4548-C95A*, *ptmT4* gene	This study
pMM-28	pCDFDuet-1-derived plasmid bearing the *smCPS*, *stt4548-C96A*, *ptmT4* gene	This study
pMM-29	pCDFDuet-1-derived plasmid bearing the *smCPS*, *stt4548-C95A/C96A*, *ptmT4* gene	This study
pMM-30	pMCSG19-derived plasmid bearing the *sat1646* gene	This study
pMM-31	pET-28a (+)-derived plasmid bearing the *stt4548* gene	This study
***E. coli* strains**		
E. coli BL21(DE3)	Heterologous host for protein production	Beijing TransGen Biotech Co., Ltd
MM-1	*E. coli* BL21(DE3) with the plasmid pMM-1 and pMM-3 transformed	This study
MM-2	*E. coli* BL21(DE3) with the plasmid pMM-1 and pMM-4 transformed	This study
MM-3	*E. coli* BL21(DE3) with the plasmid pMM-1 and pMM-5 transformed	This study
MM-4	*E. coli* BL21(DE3) with the plasmid pMM-1 and pMM-6 transformed	This study
MM-5	*E. coli* BL21(DE3) with the plasmid pMM-1 and pMM-7 transformed	This study
MM-6	*E. coli* BL21(DE3) with the plasmid pMM-1 and pMM-8 transformed	This study
MM-7	*E. coli* BL21(DE3) with the plasmid pMM-1 and pMM-9 transformed	This study
MM-8	*E. coli* BL21(DE3) with the plasmid pMM-1 and pMM-10 transformed	This study
MM-9	*E. coli* BL21(DE3) with the plasmid pMM-1 and pMM-11 transformed	This study

MM-10	*E. coli* BL21(DE3) with the plasmid pMM-1 and pMM-12 transformed	This study
MM-11	*E. coli* BL21(DE3) with the plasmid pMM-1 and pMM-13 transformed	This study
MM-12	*E. coli* BL21(DE3) with the plasmid pMM-1 and pMM-14 transformed	This study
MM-13	*E. coli* BL21(DE3) with the plasmid pMM-1 and pMM-15 transformed	This study
MM-14	*E. coli* BL21(DE3) with the plasmid pMM-1 and pMM-16 transformed	This study
MM-15	*E. coli* BL21(DE3) with the plasmid pMM-1 and pMM-17 transformed	This study
MM-16	*E. coli* BL21(DE3) with the plasmid pMM-1 and pMM-18 transformed	This study
MM-17	*E. coli* BL21(DE3) with the plasmid pMM-1 and pMM-19 transformed	This study
MM-18	*E. coli* BL21(DE3) with the plasmid pMM-1 and pMM-20 transformed	This study
MM-19	*E. coli* BL21(DE3) with the plasmid pMM-1 and pMM-21 transformed	This study
MM-20	*E. coli* BL21(DE3) with the plasmid pMM-1 and pMM-22 transformed	This study
MM-21	*E. coli* BL21(DE3) with the plasmid pMM-1 and pMM-23 transformed	This study
MM-22	*E. coli* BL21(DE3) with the plasmid pMM-1 and pMM-24 transformed	This study
MM-23	*E. coli* BL21(DE3) with the plasmid pMM-1 and pMM-25 transformed	This study
MM-24	*E. coli* BL21(DE3) with the plasmid pMM-1 and pMM-26 transformed	This study
MM-25	*E. coli* BL21(DE3) with the plasmid pMM-1 and pMM-27 transformed	This study
MM-26	*E. coli* BL21(DE3) with the plasmid pMM-1 and pMM-28 transformed	This study
MM-27	*E. coli* BL21(DE3) with the plasmid pMM-1 and pMM-29 transformed	This study
MM-28	*E. coli* BL21(DE3) with the plasmid pMM-30 transformed	This study
MM-29	*E. coli* BL21(DE3) with the plasmid pMM-31 transformed	This study

Table S4. The information of synthesized genes in the construction of engineered *E. coli* strains.

Genes	Products	Origins	Accession numbers	References
ptmT4	GGPP	*Streptomyces platensis*	AIW55562.1	[1]
smCPS	*normal*-CPP	*Salvia miltiorrhiza*	B8PQ84.1	[2]
osCPS4	*syn*-CPP	*Oryza sativa*	AY530101	[3]
ptmT2	*ent*-CPP	*Streptomyces platensis*	ACO31276	[1]
haur_2145	KPP	*Herpetosiphon aurantiacus*	ABX04785.1	[4]
kgTPS	*syn*-KPP	*Kitasatospora griseola*	BAB39206.1	[5]
mtHPS	TPP	*Mycobacterium tuberculosis*	NP_217894.1	[6]

Table S5. The ^{1}H NMR (400 MHz for **1** and 600 MHz for **2-9**) data (δ_H, *J* in Hz) of **1-9** in $CDCl_3$.

position	**1**[7]	**2**	**3**[8]	**4**[9]	**5**[9,10]	**6**[11]	**7**[8]	**8**[12]	**9**[13]
1	1.72, m; 1.00, m	1.48, m; 1.10, m	1.39, m; 1.17, m	1.64, m; 1.34, m	1.64, m; 1.34, m	1.61, m; 1.13, m	1.42, m; 1.18, m	1.81, m; 0.99, m	1.71, m; 1.01, m
2	1.66, m; 1.57, m	1.60, m; 1.41, m	1.57, m; 1.41, m	1.65, m; 1.56, m	1.65, m; 1.62, m	1.45, m; 1.38, m	1.57, m; 1.42, m	1.52, m; 1.44, m	1.5, m; 1.45, m
3	1.38, m; 1.14, m	1.40, m; 1.13, m	1.40, m; 1.13, m	1.39, m; 1.15, m	1.39, m; 1.15, m	1.11, m; 1.36, m	1.42, m; 1.16, m	1.41, m; 1.16, m	1.40, m; 1.18, m
4	-	-	-	-	-	-	-	-	-
5	1.12, m	1.34, m	1.18, m	0.95, m	0.93, m	1.23, m	1.23, m	1.12, m	1.02, m
6	1.66, m; 1.44, m	2.02, m; 1.81, m	1.97, m; 1.83, m	1.46, m; 1.29, m	1.61, m; 1.29, m	1.39, m; 1.23, m	1.99, m; 1.86, m	1.91, m; 1.87, m	1.58, m; 1.29, m
7	1.91, m	5.28, br t (6)	5.28, br d (6)	1.89, m; 1.01, m	1.96, m; 1.01, m	1.89, m; 1.19, m	5.31, br d (6)	5.36, m	2.25, m; 2.04, m
8	-	-	-	2.28, m	2.17, m	1.88, m	-	-	-
9	-	1.50, m	1.34, m	-	-	-	1.32, m	1.64, m	1.70, m
10	-	-	-	-	-	-	-	-	-
11	1.90, br s	1.89, m; 1.25, m	1.64, m; 1.25, m	5.28, br d (6)	5.29, br d (6)	1.50, m; 1.58, m	1.71, m; 1.26, m	1.53, m; 1.35, m	1.59, m; 1.52, m
12	1.49, m; 1.31, m	2.28, m; 2.05, m	1.73, m; 1.37, m	2.05, br d (16.8); 1.70, m	1.95, m; 1.34, m	2.09, m; 2.28, m	1.51, m; 1.44, m	1.49, m; 1.36, m	1.45, m; 1.35, m
13	-	-	-	-	-	-	-	-	-
14	1.79, br s; 1.73, br s;	4.70, m	2.00, m; 1.91, m	1.49, m; 1.10, m	1.59, m; 1.07, m	1.32, m; 1.78, m	2.00, m; 1.81, m	1.96, m; 1.91, m	5.21, br s

15	5.75, dd (18, 12);	2.49, m	5.91, dd (18, 12);	5.83, dd (18, 12);	5.78, dd (18, 12);	2.69, m	5.82, dd (18, 12);	5.81, dd, (18, 12);	5.78, dd (18, 12);
16	4.89, dd (18, 2.4); 4.85, dd (12; 2.4)	2.28, m; 2.02, m	4.95, dd (12, 1.5); 4.94, dd (18, 1.5) ;	4.94, d (18); 4.87, d (12)	4.89, dd (18, 1.1); 4.88, dd (12, 1.1)	1.20, m; 2.09, m	4.93, dd (18, 1.4); 4.86, dd (12, 1.4)	4.93, dd (18, 1.3); 4.86, dd (12, 1.3)	4.91, dd (18, 1.4); 4.88, dd (12, 1.4)
17	0.97, s	1.05, d (6)	1.00, s	0.92, s	0.98, s	4.44, br t (2.4); 4.36, br t (2.4)	0.90, s	0.87, s	1.04, s
18	0.88, s	0.85, s	0.87, s	0.85, s	0.84, s	0.88, s	0.89, s	0.87, s	0.88, s
19	0.84, s	0.88, s	0.89, s	0.86, s	0.84, s	0.87, s	0.91, s	0.92, s	0.85, s
20	0.96, s	0.88, s	0.94, s	1.04, s	0.95, s	0.97, s	0.94, s	0.87, s	0.79, s

Table S6. The ^{13}C NMR (100 MHz for **1** and 150 MHz for **2-9**) data (δ_C, type) of **1-9** in $CDCl_3$.

position	**1**[7]	**2**	**3**[8]	**4**[9]	**5**[9]	**6**[11]	**7**[14]	**8**[12,15]	**9**[13]
1	36.8, CH_2	35.9, CH_2	37.0, CH_2	37.8, CH_2	37.3, CH_2	36.2, CH_2	36.9, CH_2	40.0, CH_2	39.6, CH_2
2	19.1, CH_2	19.0, CH_2	19.0, CH_2	19.2, CH_2	19.2, CH_2	19.0, CH_2	18.9, CH_2	19.0, CH_2	19.2, CH_2
3	42.0, CH_2	43.1, CH_2	43.2, CH_2	42.5, CH_2	42.5, CH_2	42.0, CH_2	43.2, CH_2	42.4, CH_2	42.3, CH_2
4	33.5, C	33.1, C	33.0, C	33.8, C	33.8, C	33.4, C	33.0, C	33.0, C	33.5, C
5	52.0, CH	41.6, CH	43.6, CH	53.9, CH	53.8, CH	47.5, CH	43.7, CH	50.6, CH	55.0, CH
6	19.2, CH_2	24.7, CH_2	23.9, CH_2	22.1, CH_2	22.1, CH_2	22.6, CH_2	23.9, CH_2	23.6, CH_2	22.8, CH_2
7	32.7, CH_2	122.5, CH	119.7, CH	36.4, CH_2	36.2, CH_2	36.8, CH_2	120.1, CH	121.8, CH	36.2, CH_2
8	124.4, C	137.8, C	137.0, C	31.3, CH	31.9, CH	39.3, CH	136.9, C	135.7, C	137.5, C
9	137.3, C	52.6, CH	53.3, CH	149.9, C	150.7, C	51.3, C	53.3, CH	52.2, CH	50.8, CH
10	37.7, C	36.4, C	36.2, C	39.5, C	39.4, C	38.7, C	35.3, C	35.6, C	38.5, C
11	21.3, CH_2	31.8, CH_2	25.5, CH_2	112.6, CH	112.8, CH	32.3, CH_2	25.1, CH_2	20.4, CH_2	18.9, CH_2
12	35.2, CH_2	34.2, CH_2	39.7, CH_2	37.4, CH_2	37.8, CH_2	28.4, CH_2	38.0, CH_2	36.4, CH_2	34.8, CH_2
13	35.2, C	155.5, C	39.0, C	34.9, C	35.3, C	155.9, C	39.0, C	37.0, C	37.6, C
14	42.2, CH_2	108.7, CH	49.8, CH_2	42.8, CH_2	44.5, CH_2	39.9, CH_2	48.2, CH_2	46.3, CH_2	128.6, CH
15	146.5, CH	37.2, CH	146.0, CH	150.5, CH	145.7, CH	43.6, CH	150.7, CH	150.7, CH	149.4, CH
16	110.8, CH_2	45.7, CH_2	111.2, CH_2	109.3, CH_2	110.9, CH_2	37.8, CH_2	109.2, CH_2	109.3, CH_2	110.1, CH_2
17	28.0, CH_3	19.5, CH_3	29.8, CH_3	22.1, CH_3	29.3, CH_3	101.7, CH_3	21.9, CH_3	21.6, CH_3	26.1, CH_3
18	33.4, CH_3	33.1, CH_3	33.6, CH_3	33.5, CH_3	33.5, CH_3	34.7, CH_3	33.6, CH_3	33.8, CH_3	33.9, CH_3
19	21.9, CH_3	22.1, CH_3	22.8, CH_3	22.1, CH_3	22.1, CH_3	23.0, CH_3	22.9, CH_3	22.5, CH_3	22.3, CH_3
20	19.6, CH_3	21.9, CH_3	22.3, CH_3	21.1, CH_3	21.0, CH_3	18.9, CH_3	22.3, CH_3	15.1, CH_3	15.2, CH_3

Table S7. The data collection and refinement statistics of crystal structures.

	**Sat1646-Mg^{2+}**	**Sat1646**	**Stt4548**
Data collection			
Wavelength (Å)	0.97918	0.97918	0.97918
Space group	$P\ 4_2\ 2_1\ 2$	$P\ 4_2\ 2_1\ 2$	*C* 1 2 1
Unit cell			
a, b, c (Å)	89.017, 89.017, 68.674	89.013, 89.013, 70.634	66.870, 64.908, 70.583
α, β, γ (°)	90.000, 90.000, 90.000	90.000, 90.000, 90.000	90.000, 108.751, 90.000
Resolution range (Å)	62.95-1.94 (1.99-1.94)[b]	40.00-2.50 (2.59-2.50)[b]	66.84-1.58 (1.68-1.58)
Unique reflections	18106 (905)	10288 (1001)	31104 (1555)
R_{merge} [a]	0.111 (1.625)	0.084 (0.515)	0.067 (0.952)
$I/\sigma I$	18 (1.5)	45 (4.8)	13.4 (1.2)
Completeness (%)	87 (63.8)	100 (100)	90.0 (57.7)
Average redundancy	21.5 (9.4)	25.8 (25.9)	6.3 (4.9)
Structure refinement			
Resolution range (Å)	28.61-1.94	27.36-2.50	66.84-1.57
*R*work/*R*free	0.1953/0.2473	0.2228/0.2759	0.1788/0.2089
Number of protein atoms	2273	2273	2061
Number of water atoms	140	67	151
Bond lengths RMSD (Å)	0.007	0.008	0.018
Bond angles RMSD (°)	0.844	0.912	1.852
Average B-factors (Å^2)	39.0958	71.4443	35.5700
Ramachandran plot (%)			
Most favored	96.89	94.81	98.12
Additional allowed	1.73	3.81	1.5
Outliers	1.38	1.38	0.38
Protein Data Bank entry	7E4N	7E4O	7E4M

[a]$R_{merge} = \sum_{hkl}\sum_i |I_i(hkl) - \langle I(hkl)\rangle| / \sum_{hkl}\sum_i I_i(hkl)$, where $I_i(hkl)$ is the *i*th observation of reflection *hkl*, and $\langle I(hkl)\rangle$ is the weighted average intensity for all observations of that reflection *hkl*.
[b]Numbers in parentheses are values for the highest-resolution bin.

Figure S1. Representative pimarane-type diterpenoids.[16-34] including five skeletons (red) with different absolute configurations. The biological activities and producing hosts are shown in parentheses, and the producing hosts are labelled in blue for microorganisms, green for plants, purple for animals, respectively.

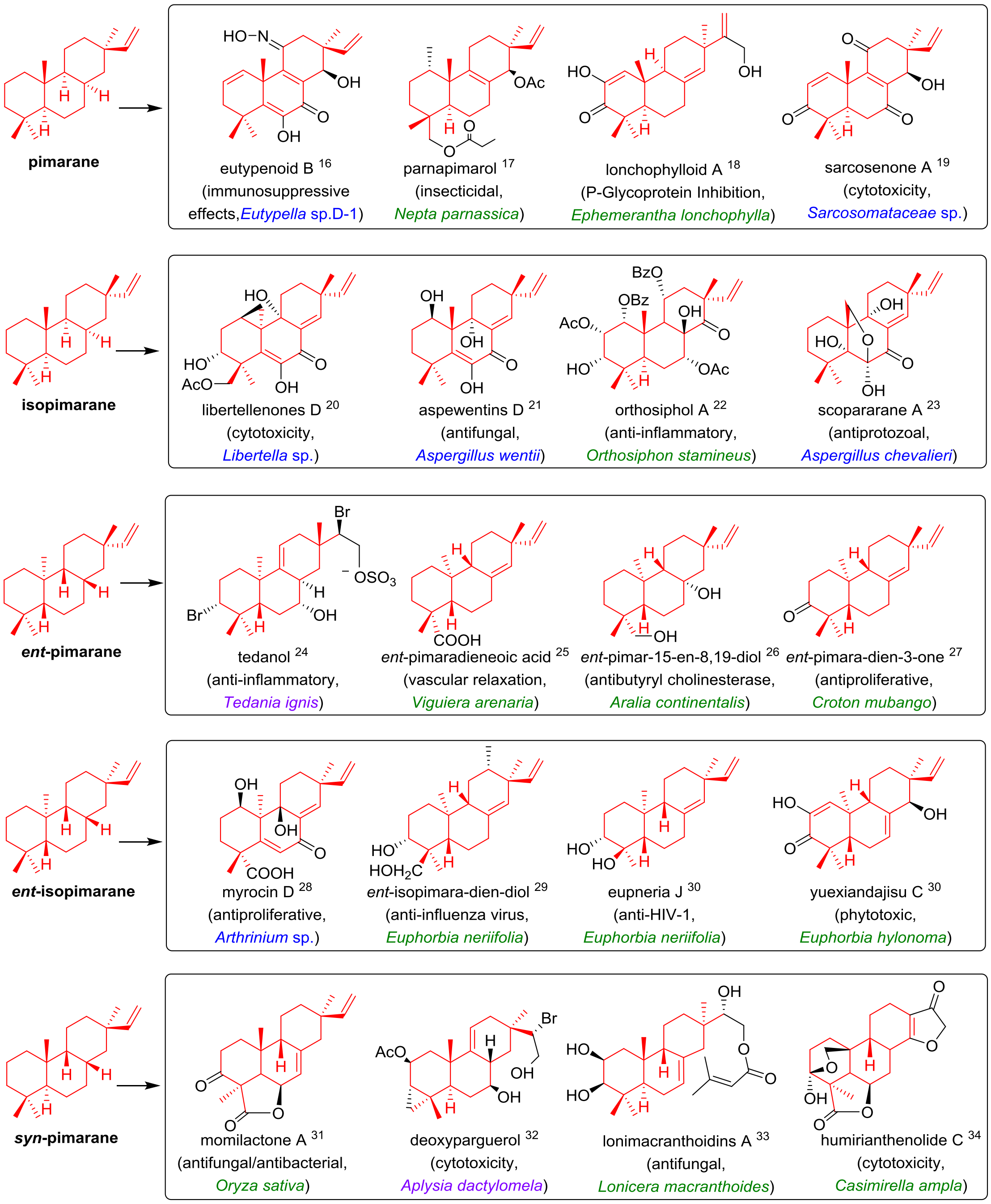

Figure S2. The reported structures of DTSs and their catalytic reactions. (A) The reported structures of DTSs. Class I DTSs include LrdC,[35] BjKS,[36] CotB2,[37] PaFS$_{344}$,[38] FgGS,[39] SvS-A2[40] and Rv3378c[41] that only contain the α-domain, and TXS[42] that contains α, β and γ domains but only the α domain is functional. Class II DTSs include PtmT2[43] that contains β and γ domains, and AtCPS[44] that contains α, β and γ domains but the α domain is nonfunctional. The class I+II DTS AgAS[45] contains functional α, β and γ domains. Structures are colored from blue on the N-terminus to red on the C-terminus. The DDxxD and NSE (class I), DxDD (class II) motifs are shown in black, and the active sites are highlighted with red dashed circles. The PDB IDs are shown in parentheses. (B) The catalytic reactions of DTSs. The product of Sat1646 or Stt4548 is highlighted in red.

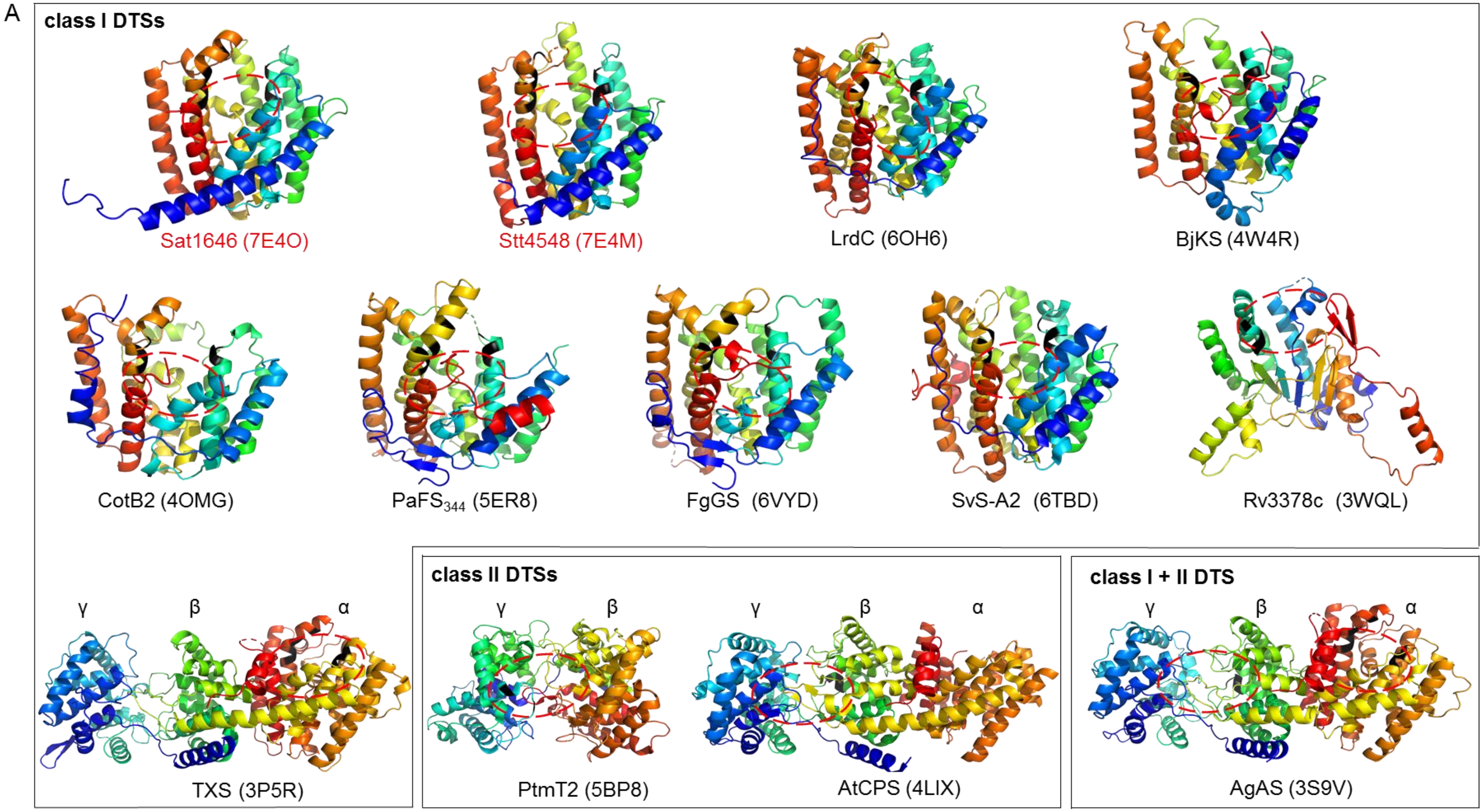

B

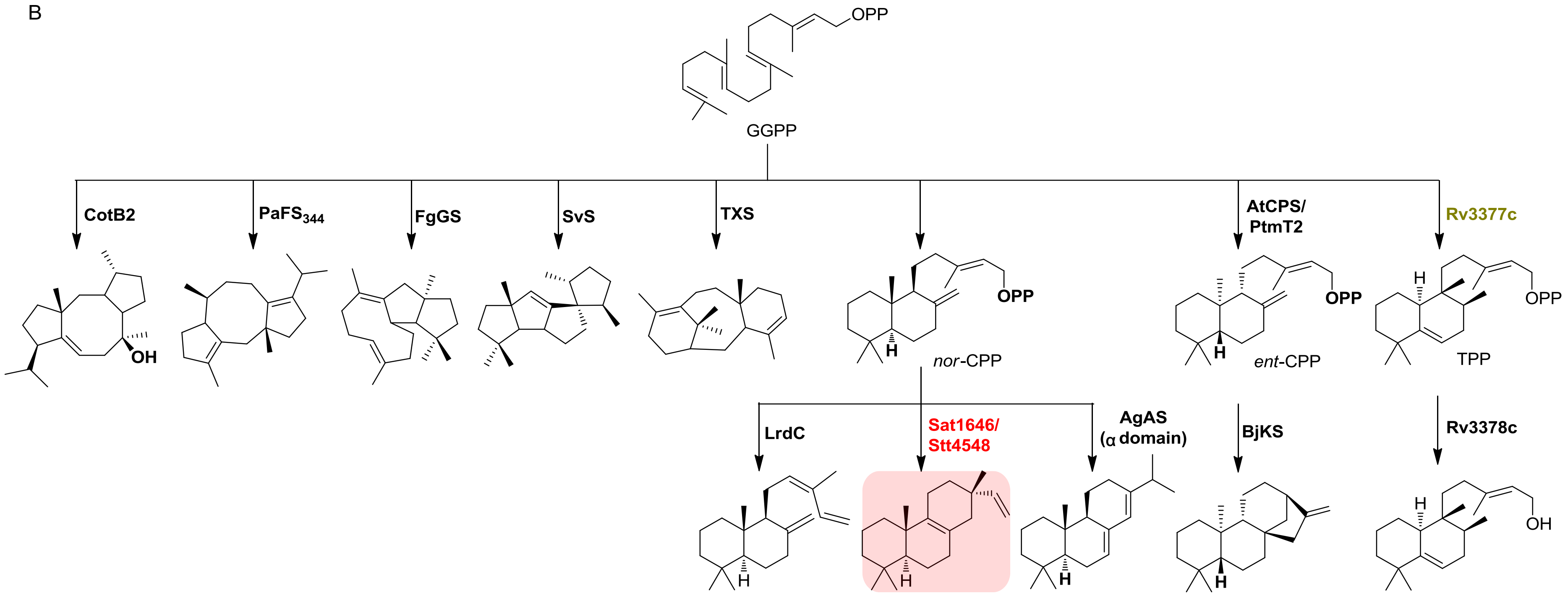

OPP
GGPP
CotB2
OH
PaFS344
FgGS
SvS
TXS
OPP
H
nor-CPP
AtCPS/
PtmT2
OPP
H
ent-CPP
Rv3377c
H
OPP
TPP
LrdC
H
Sat1646/
Stt4548
H
AgAS
(α domain)
H
BjKS
H
Rv3378c
H
OH

Figure S3. The degenerate primers designed for genome mining.

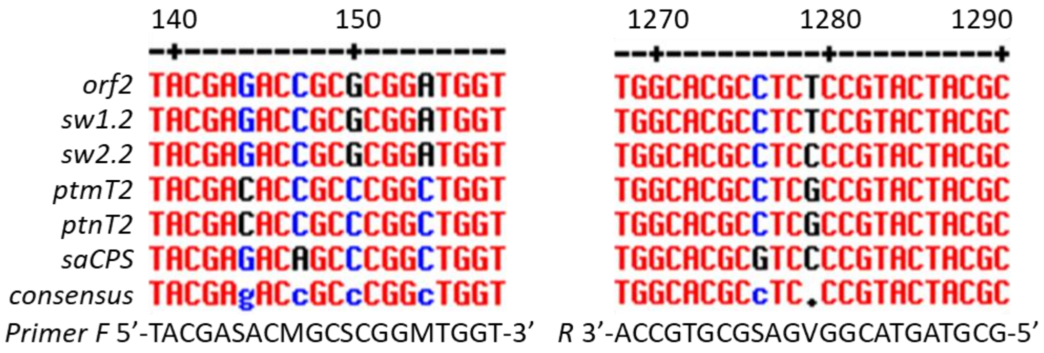

Figure S4. The phylogenetic analysis of strains PKU-MA00418 and PKU-TA00600 (labelled in red) based on the comparison of 16S rRNA sequences. The GenBank accession numbers are shown in parentheses. The 16S rRNA sequence of *Salinispora arenicola* atrsin PTM-099 was used as an outgroup for the phylogenetic analysis of strain PKU-TA00600.

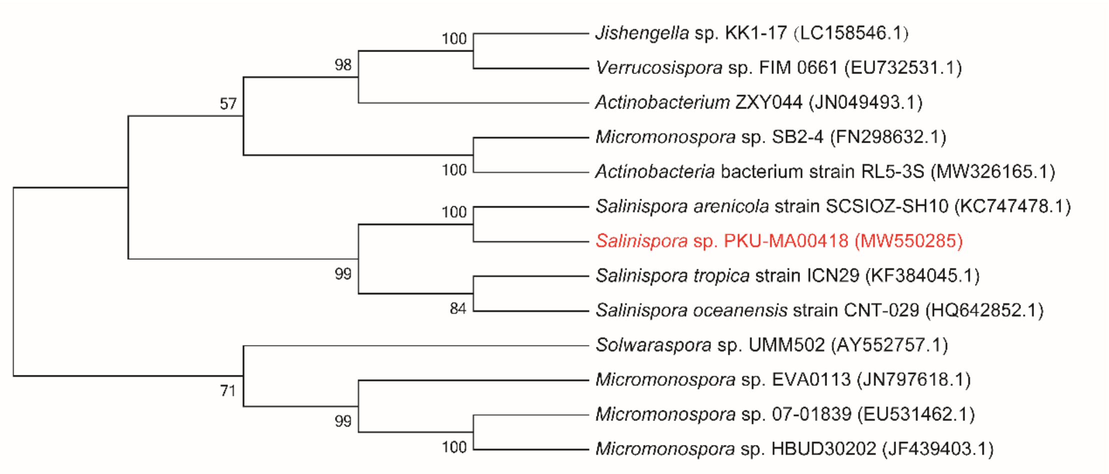

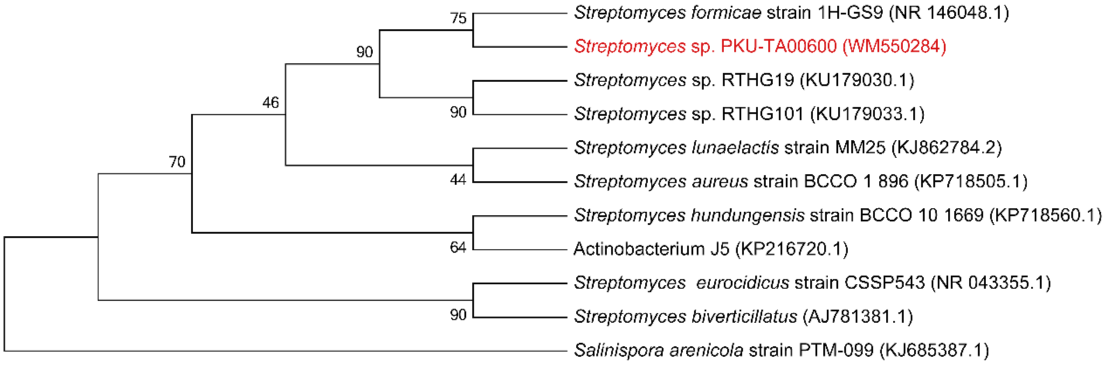

Figure S5. Comparison between *sat* gene cluster and *terp1* gene cluster. (A) The comparison of *sat* gene cluster from *Salinispora* sp. PKU-MA00418 and *terp1* cluster from *Salinispora arenicola* CNS-205. (B) The sequence alignment of Sat1645 with SaCPS. (C) The sequence alignment of Sat1646 with SaDTS. (D) The sequence alignment of Sat1647 with CYP1051A1.

A

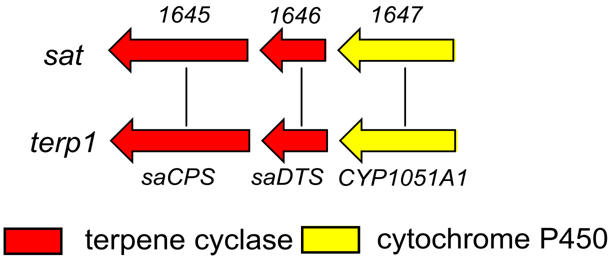

B

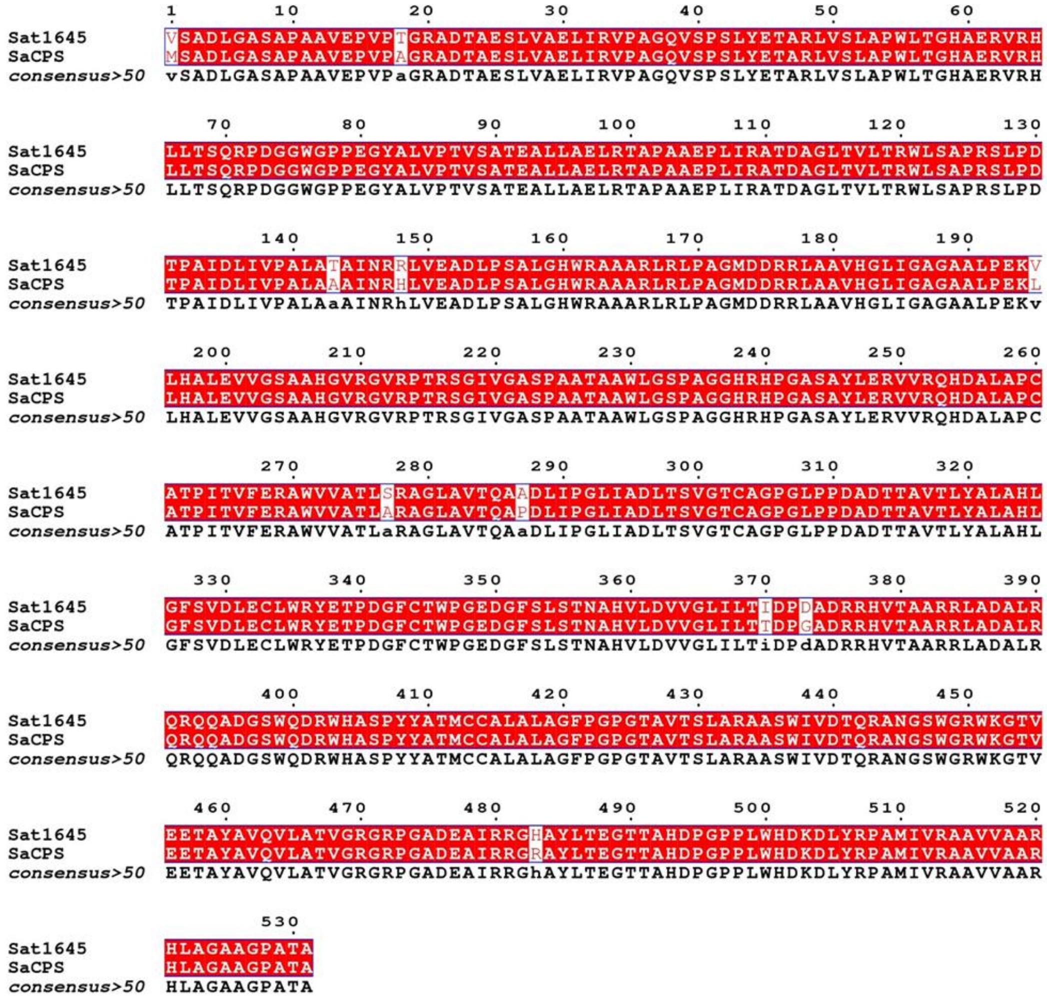

C

```
               1        10        20        30        40        50        60
Sat1646        VPTHHAGNGFAVASEQGRICALAARGQRDLRQCVRAYPSLFPNPPVDDTMLSALALSTAFIAPWC
SaDTS          MPTHHAGNGFAVASEQGRICALAARGQRDLRQCVRAYPSLFPNPPVDDTMLSALALSTAFIAPWC
consensus>50   vPTHHAGNGFAVASEQGRICALAARGQRDLRQCVRAYPSLFPNPPVDDTMLSALALSTAFIAPWC

                    70        80        90       100       110       120       130
Sat1646        SAEQLRVANRASLWVTAEDWQVDRVATSDDAVRSIVSACQAVADGAAPDVDCALGQLLAEIRDEL
SaDTS          SAEQLRVANRASLWVTAEDWQVDRVATSDDAVRSIVSACQAVADGAAPDVDCALGQLLAEIRDEL
consensus>50   SAEQLRVANRASLWVTAEDWQVDRVATSDDAVRSIVSACQAVADGAAPDVDCALGQLLAEIRDEL

                         140       150       160       170       180       190
Sat1646        ATGAGFTEWQPVWREEVRRMLTADIREWEWRHSARPPSFAEYLDNADNYGASFVNVSHWIVTGDA
SaDTS          ATGAGFTEWQPVWREEVHRMLTADIREWEWRHSARPPSFAEYLDNADNYGASFVNVSHWIVTGDA
consensus>50   ATGAGFTEWQPVWREEVhRMLTADIREWEWRHSARPPSFAEYLDNADNYGASFVNVSHWIVTGDA

                  200       210       220       230       240       250       260
Sat1646        QTRSHLPELIAASREVQRILRLSNDLASYERDIRSGDLNALLLVDREEVSRQLRDRIRACQDHLH
SaDTS          QTRSHLPELIAASREVQRILRLSNDLASYERDIRSGDLNALLLVDREEVSRQLRDGIRACQDHLH
consensus>50   QTRSHLPELIAASREVQRILRLSNDLASYERDIRSGDLNALLLVDREEVSRQLRDgIRACQDHLH

                         270       280       290
Sat1646        ALEVTCPREALYLAREAGFTTGFYHGADYWGDSER
SaDTS          TLEVTCPREALYLAREAGFTTGFYHGADYWGDSER
consensus>50   aLEVTCPREALYLAREAGFTTGFYHGADYWGDSER
```

D

```
               1        10        20        30        40        50        60
Sat1647        VPAWKALPAAVRDTHRALVDVGNWSDGDVVQVSLGVSRPYLVTNPAHVQEVLHERAAIYPRGDDT
CYP1051A1      MPAWKALPAAVRDTHRAFVDVGNWSDGDVVRVSLGVSRPYLVTNPAHVQEVLHERAAIYPRGDDT
consensus>50   vPAWKALPAAVRDTHRAlVDVGNWSDGDVVqVSLGVSRPYLVTNPAHVQEVLHERAAIYPRGDDT

                    70        80        90       100       110       120       130
Sat1647        ALWRSVRKLVGDGILAEGDAWAASRRVLAPMFRPARINAMVDTMADAIAGAVDDLHGAATAGTPI
CYP1051A1      ALWRSVRKLVGDGILAEGDAWAASRRVLAPMFRPARINAMVDTMADAIAGAIDDLHGAATAGTPI
consensus>50   ALWRSVRKLVGDGILAEGDAWAASRRVLAPMFRPARINAMVDTMADAIAGA!DDLHGAATAGTPI

                         140       150       160       170       180       190
Sat1647        DVGRELSRIVCSAIMRVFFADRIAVRDALRIMKAQETIVTAMAPRILAPLVPWWIPMPGDRRFRA
CYP1051A1      DVGRELSRIVCSAIMRVFFADRITVRDALRIMKAQETIVTAMAPRILAPLVPWWIPMPGDRRFRA
consensus>50   DVGRELSRIVCSAIMRVFFADRIaVRDALRIMKAQETIVTAMAPRILAPLVPWWIPMPGDRRFRA

                  200       210       220       230       240       250       260
Sat1647        AVRSIDDILLPVLRQAQRQPDDGDDLLSRLVRARADDGRALSEKRMRDDLVSMVAVTTETSTVVL
CYP1051A1      AVRSIDDILLPVLRQAQRQPDDGDDLLSRLVHARADDGRALSEKRMRDDLVSMVAVTTETSTVVL
consensus>50   AVRSIDDILLPVLRQAQRQPDDGDDLLSRLVhARADDGRALSEKRMRDDLVSMVAVTTETSTVVL

                         270       280       290       300       310       320
Sat1647        TWLWPLLANHPDVANRLYDEIDRVVGGGPVRGDHLAELTYTRMVLDELLRLYPAGWILPRRAATT
CYP1051A1      TWLWPLLANHPDVANRLYDEIDRVVGGGPVRGDHLAELTYTRMVLDELLRLYPAGWILPRRAATT
consensus>50   TWLWPLLANHPDVANRLYDEIDRVVGGGPVRGDHLAELTYTRMVLDELLRLYPAGWILPRRAATT

                  330       340       350       360       370       380       390
Sat1647        DVLGGVRINKGATVILSPYVTQRMTAWWGPTAEAFDPERFASGREAADGRHRYAYYPFGVGMHRC
CYP1051A1      DILGGVRINKGATVILSPYVTQRMTAWWGPTAEAFDPERFAAGREAADGRHRYAYYPFGVGMHRC
consensus>50   D!LGGVRINKGATVILSPYVTQRMTAWWGPTAEAFDPERFAaGREAADGRHRYAYYPFGVGMHRC

                       400       410       420       430       440
Sat1647        LGEHLFNLEAILIVATLLSRFRFALTDTSMPGVKVAASTRPARTVELVLKPVAPVPAR
CYP1051A1      LGEHLFNLEAILIVATLLSRFRFALTDTSMPGVKVAASTRPARTVEVILKPVAPVPAR
consensus>50   LGEHLFNLEAILIVATLLSRFRFALTDTSMPGVKVAASTRPARTVEv!LKPVAPVPAR
```

Figure S6. The construction of engineered *E. coli* strains bearing terpene precursor genes and DTSs-encoding genes.

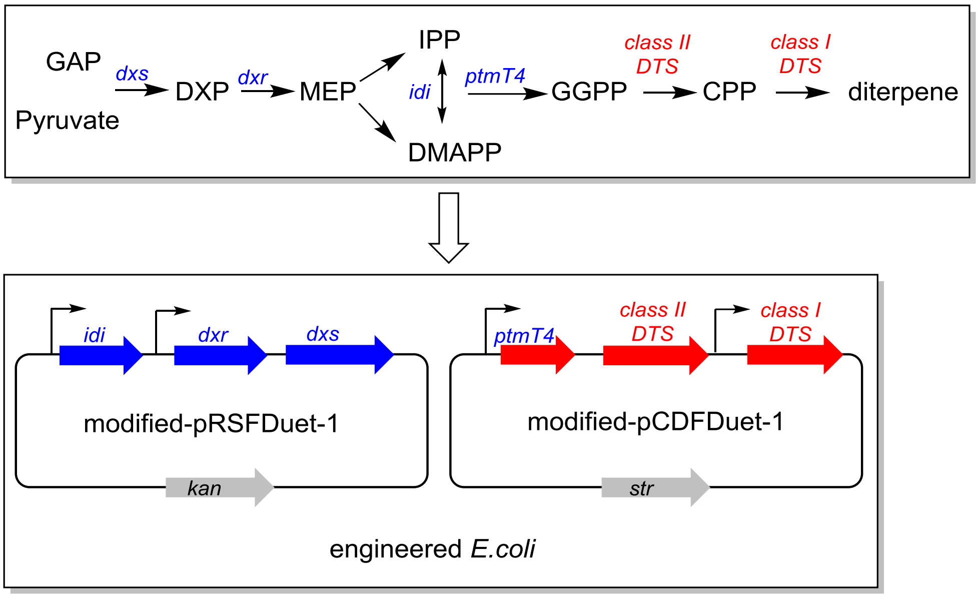

Figure S7. Mass spectra of compounds **1-9** identified from GC-MS analysis.

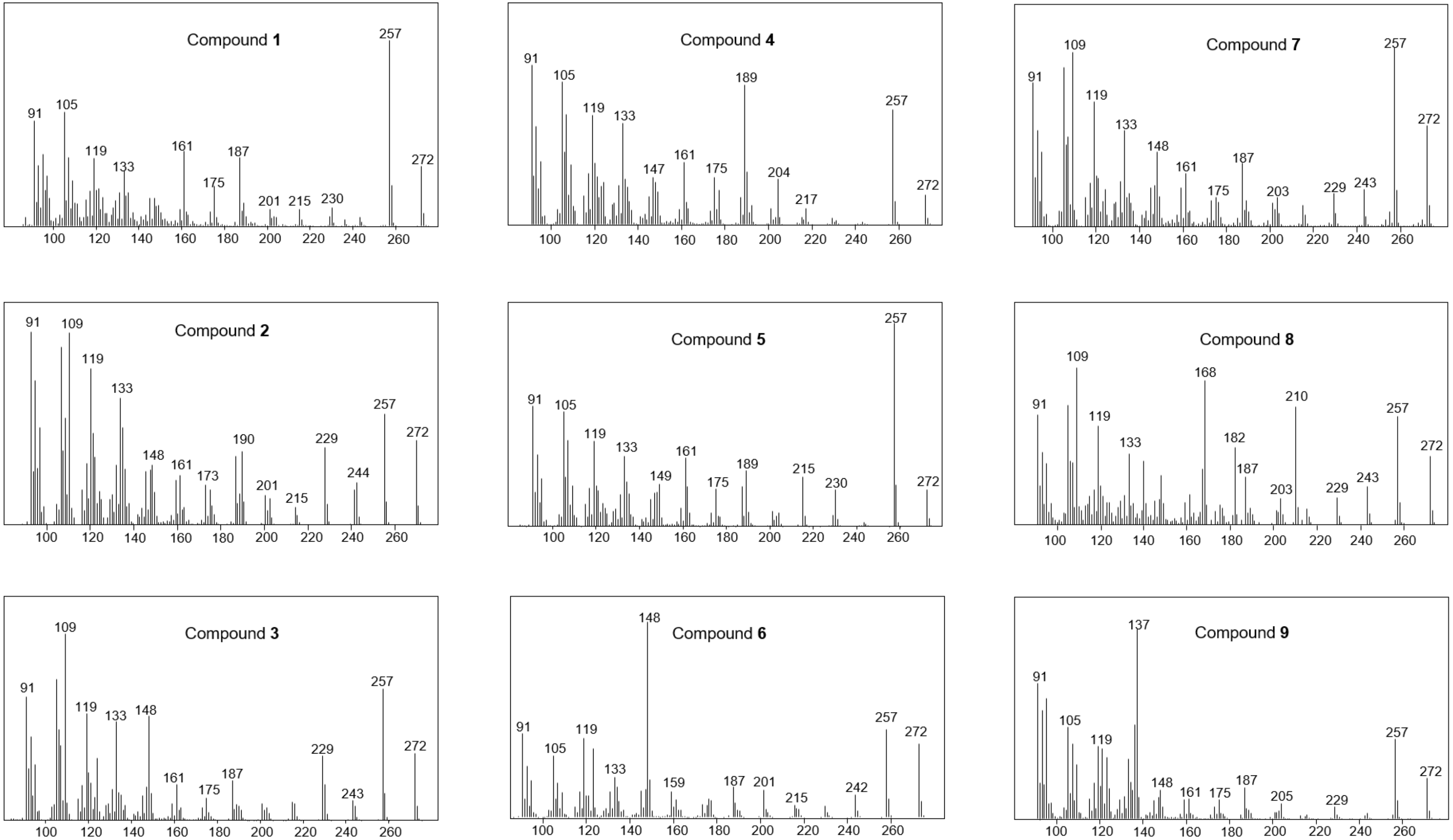

Figure S8. The SDS-PAGE of Sat1646 and Stt4548, and the TLC analysis of compound **2**. (A) The SDS-PAGE of Sat1646 and Stt4548. The calculated molecule weight of Sat1646: 33.3 kDa, and the calculated molecule weight of Stt4548: 33.7 kDa. (B) The TLC analysis of compound **2**, eluted with petroleum ether and visualized by anisaldehyde-sulphuric acid reagent.

A

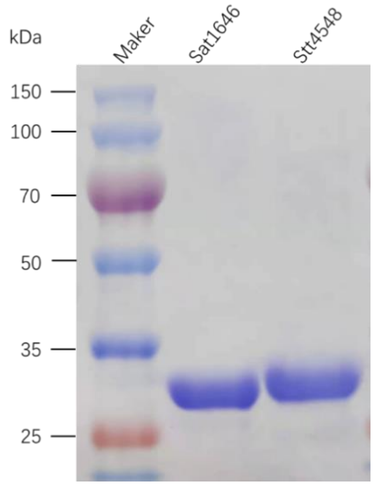

B

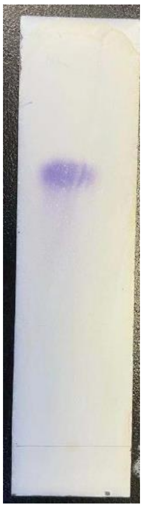

Figure S9. The crystallographic packing of Sat1646-Mg^{2+}. (A) The crystallographic packing of Sat1646-Mg^{2+}. Three molecules were shown in magenta (the first molecule), yellow (the second molecule) and gray (the third molecule), respectively. (B) The amplified region of the N-terminal loop from the first molecule interacting with Arg230 from the third molecule. (C) The position of Asn2 from the N-terminal loop of the first molecule in the active site of the third molecule. The residues from the N-terminal loop of the first molecule were shown in magenta sticks. The magnesium ions of the third molecule were shown in green spheres, and the residues from the two motifs of the third molecule were shown in gray sticks.

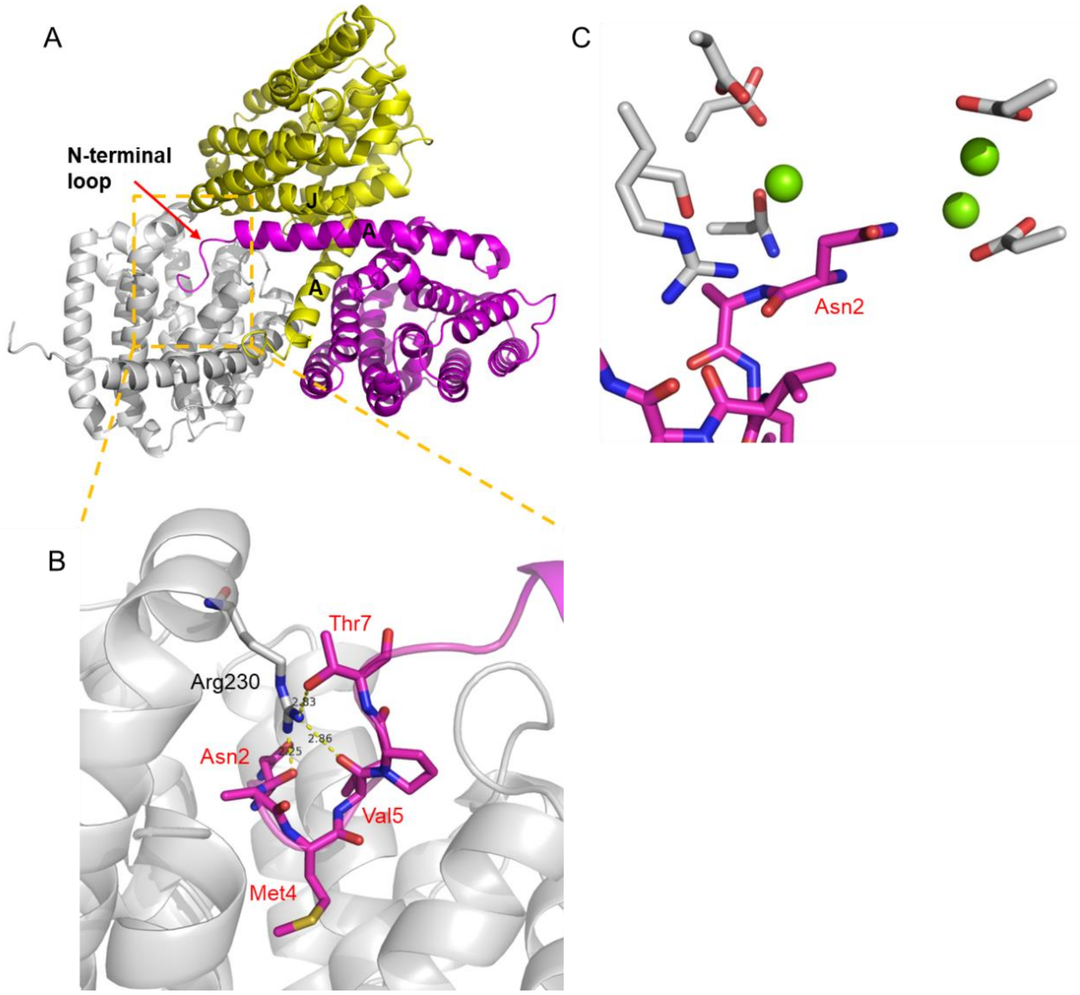

Figure S10. The comparison between Sat1646-Mg^{2+} and apo-Sat1646 in general structures, motifs and B factor values. (A) The superimposition of Mg^{2+}-binding motifs of Sat1646-Mg^{2+} (residues in gray sticks and Mg^{2+} ions in green spheres) and Sat1646 (residues in cyan). (B) The structural superimposition of Sat1646-Mg^{2+} (gray) and apo-Sat1646 (cyan). (C) Comparison between Sat1646-Mg^{2+} and apo-Sat1646 in B factor values. The small to large B factor values of residues were shown from blue to red. Largest B factor values were observed around the active sit entrance of apo-Sat1646, including the residues (sticks) of the two motifs.

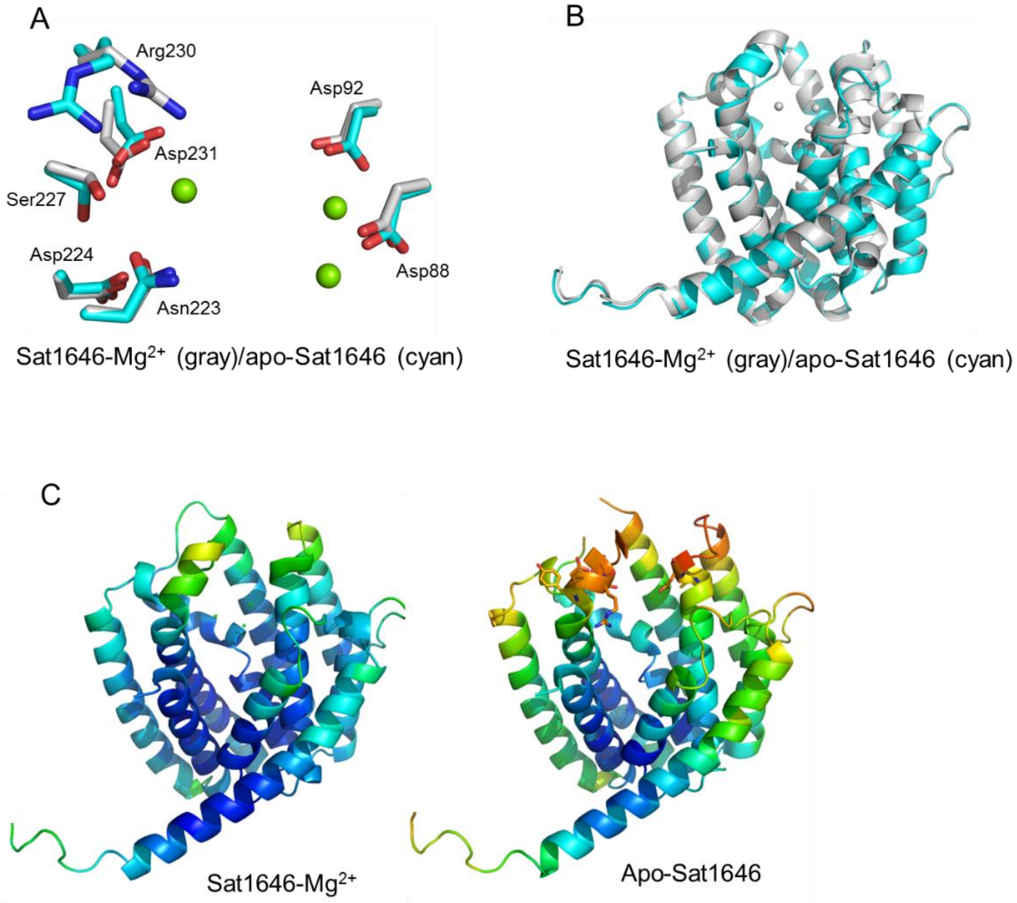

Figure S11. The crystallographic packing of Stt4548 and comparison between Stt4548 and Sat1646-Mg^{2+}. (A) The crystallographic packing of Stt4548. Two molecules were shown in magenta and green, respectively. (B) The structural superimposition of Stt4548 (magenta) and Sat1646-Mg^{2+} (gray). (C) The superimposition of Mg^{2+}-binding motifs of Sat1646-Mg^{2+} (residues in gray sticks and Mg^{2+} ions in green spheres) and Stt4548 (residues in magenta).

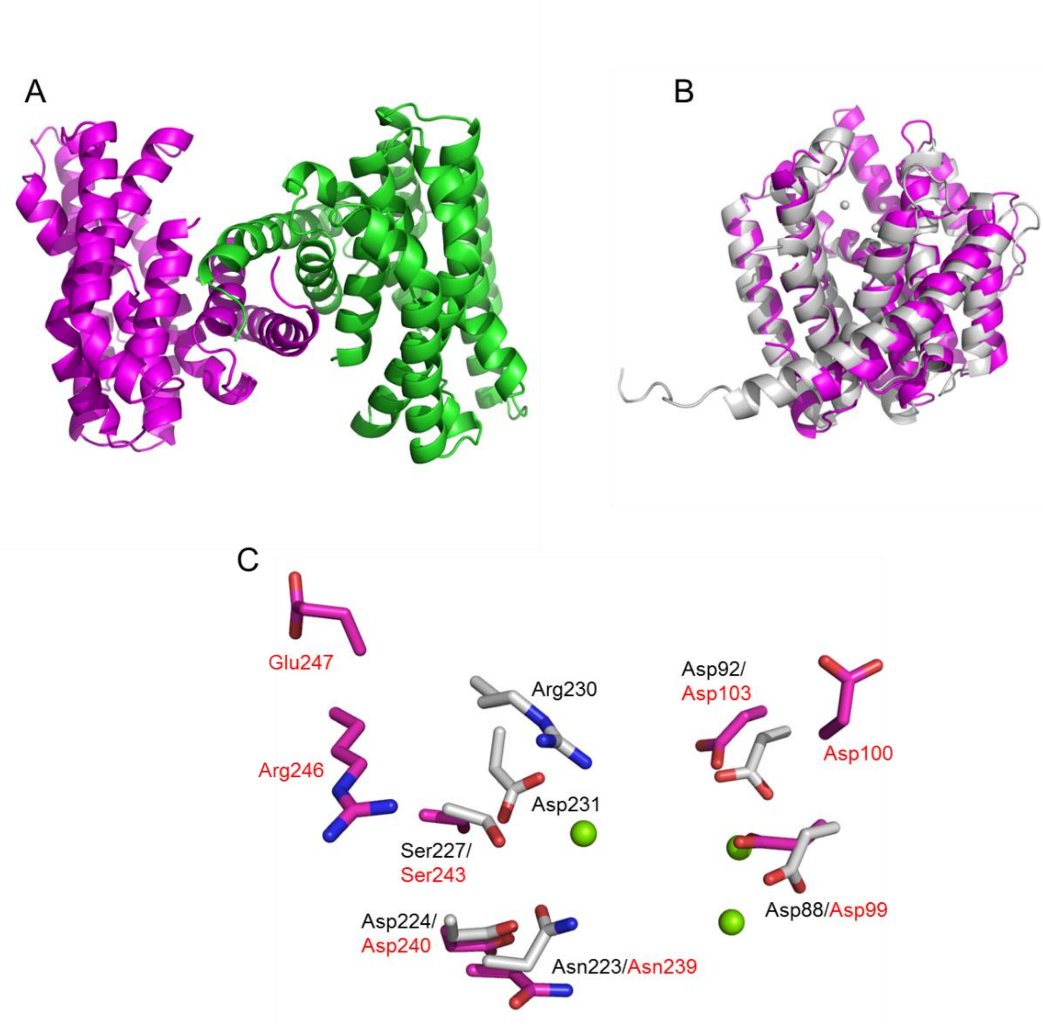

Figure S12. GC-MS analysis (extracted ion chromatogram at *m/z* 272) of the products of Sat1646 mutants with SmCPS.

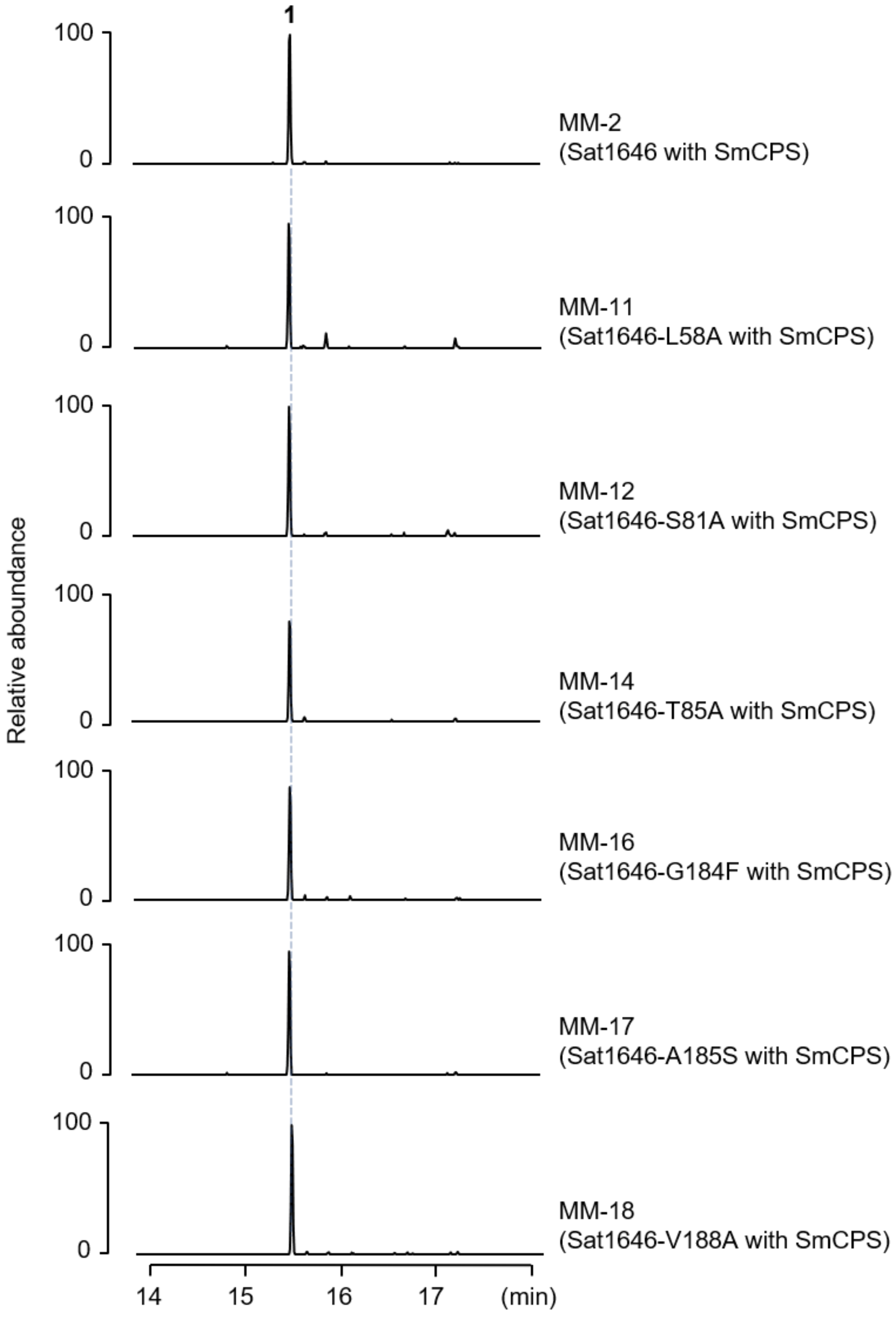

Figure S13. GC-MS analysis (extracted ion chromatogram at *m/z* 272) of the products of Stt4548 mutants with SmCPS.

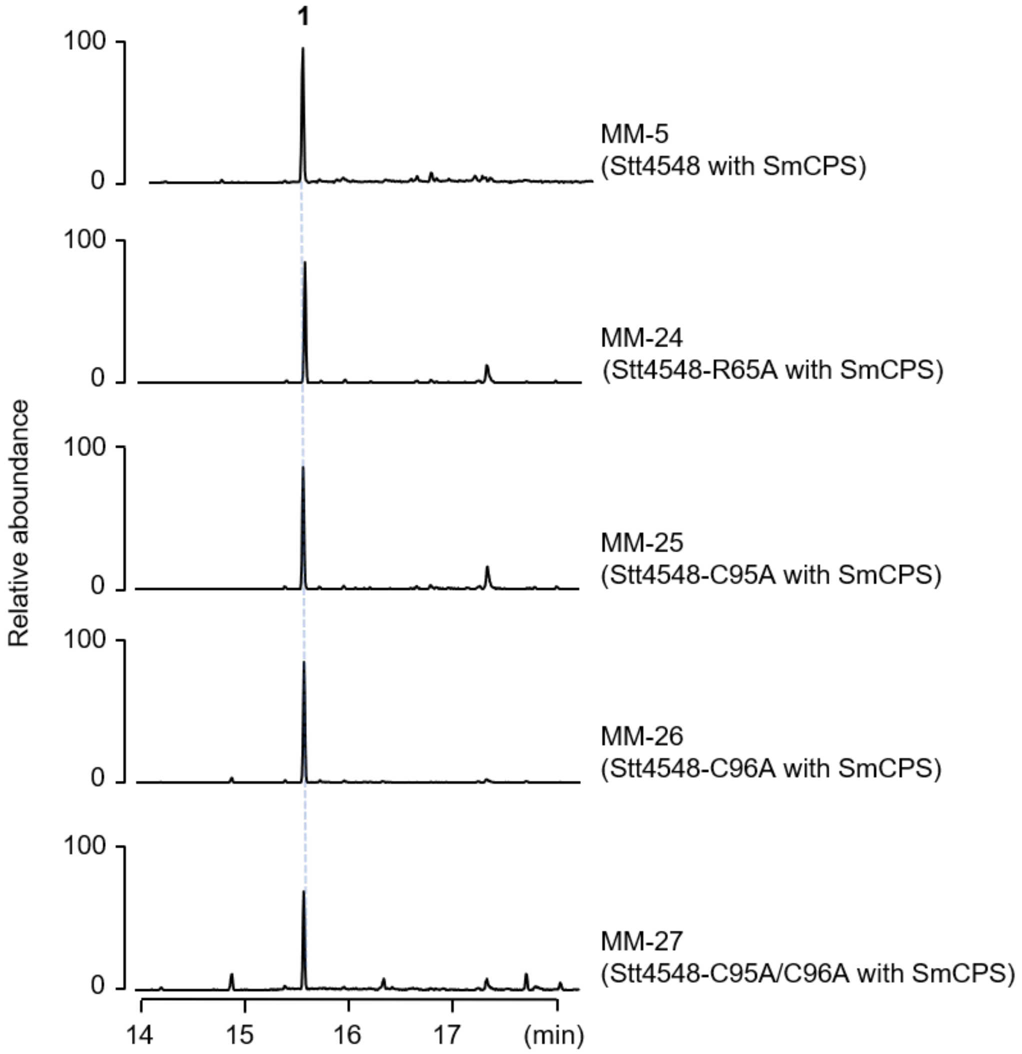

Figure S14. The proposed biosynthetic pathways of **2**-**7** (shown in black squares). The catalytic residues are shown in red.

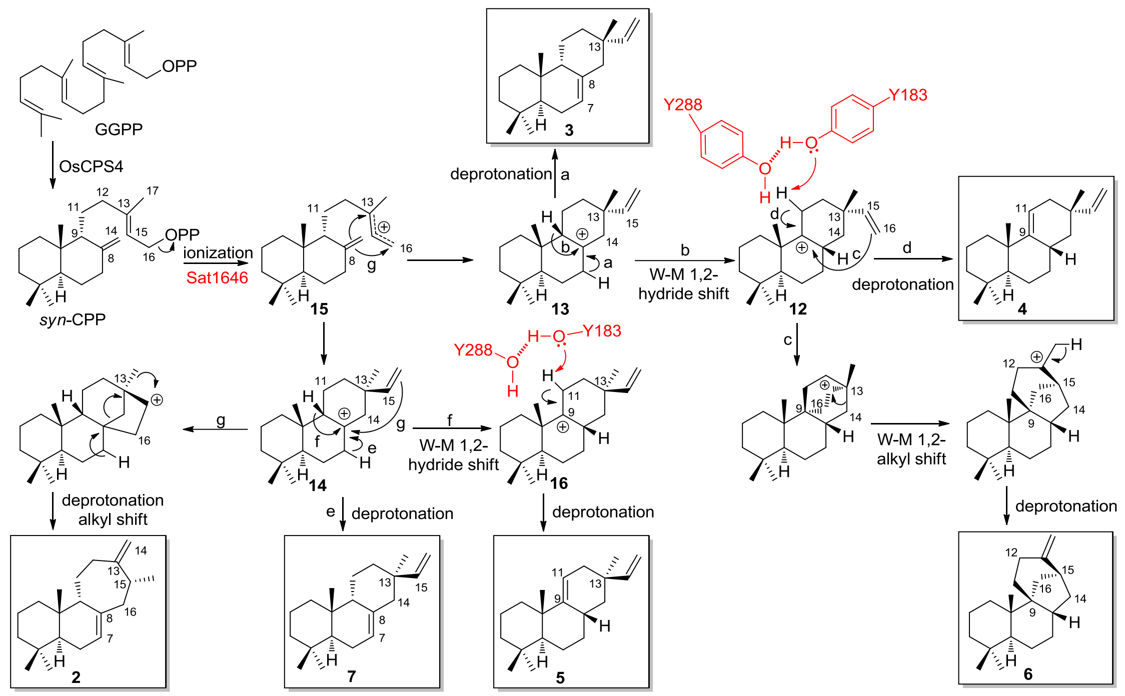

Figure S15. The ^{1}H NMR (400 MHz) and ^{13}C NMR (100 MHz) spectrum of compound **1** in $CDCl_3$.

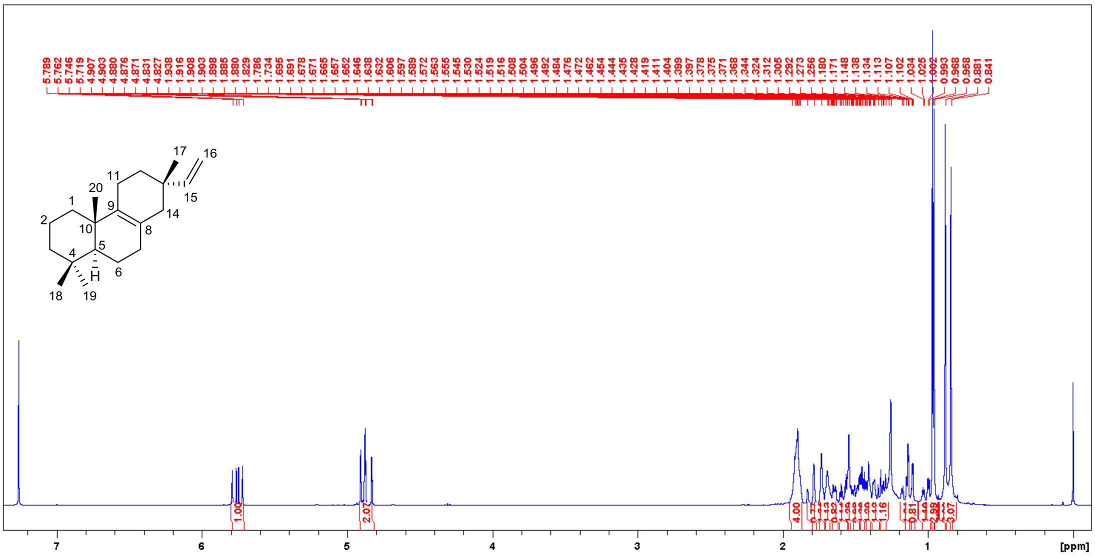

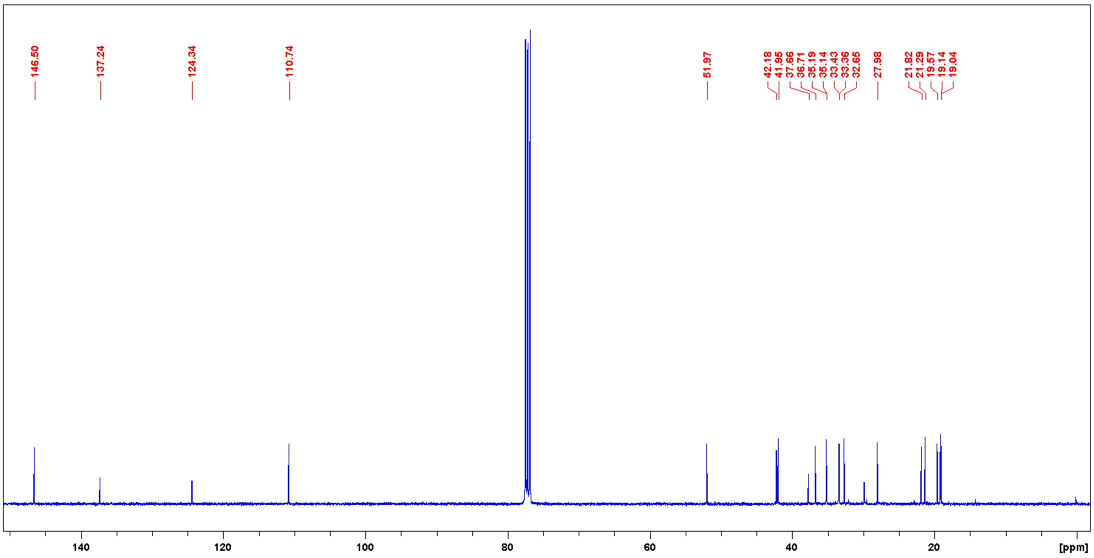

Figure S16. The ^{1}H NMR (600 MHz) and ^{13}C NMR (150 MHz) spectrum of compound **2** in $CDCl_3$.

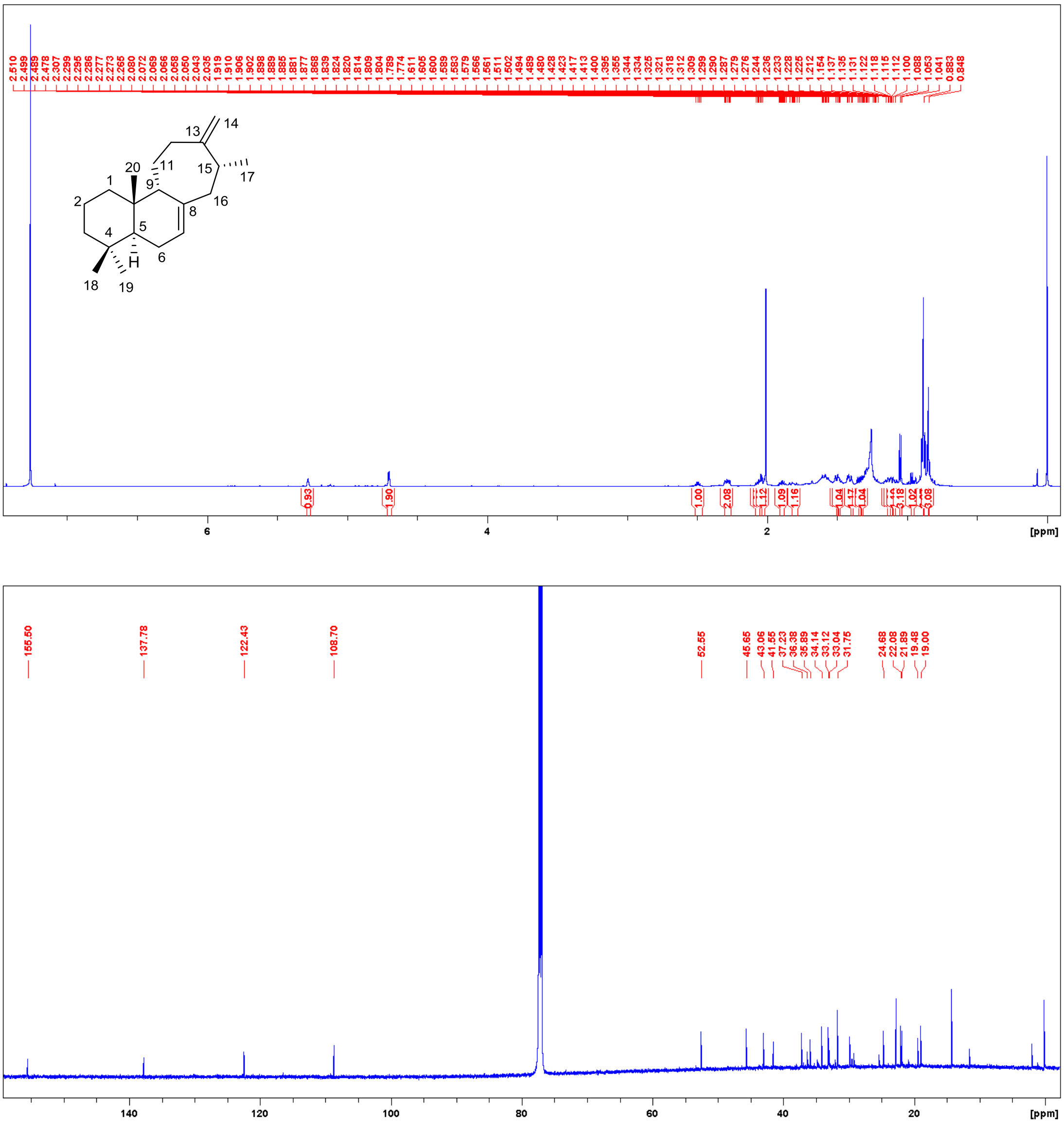

Figure S17. The COSY spectrum of compound **2** in $CDCl_3$.

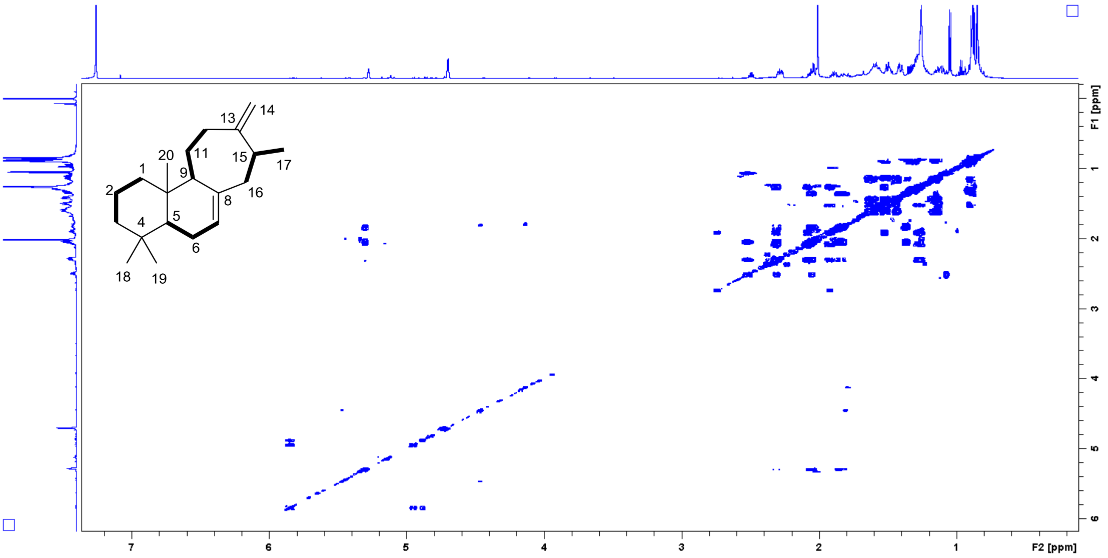

Figure S18. The HSQC spectrum of compound **2** in $CDCl_3$.

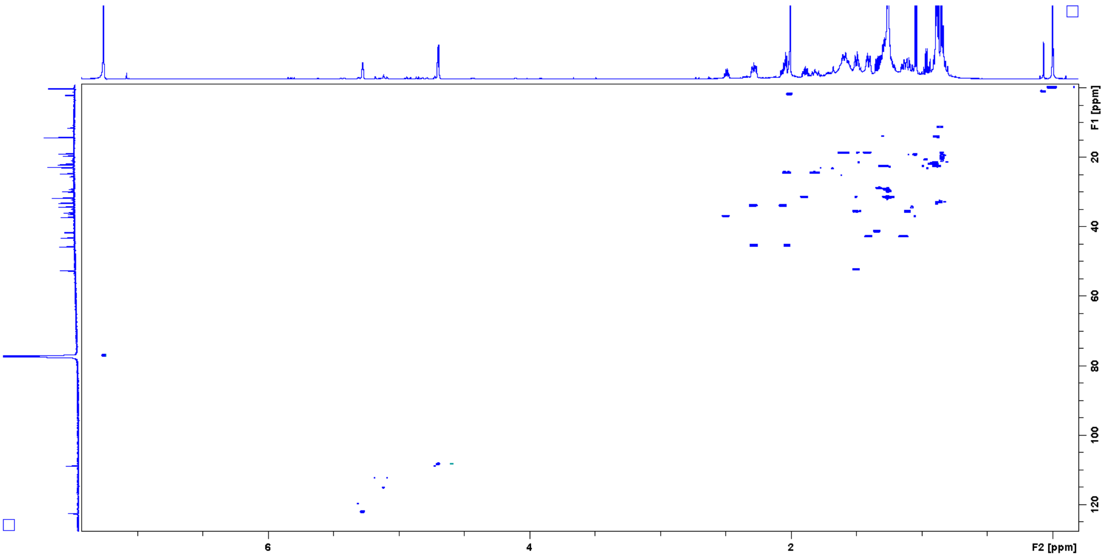

Figure S19. The HMBC spectrum of compound **2** in $CDCl_3$.

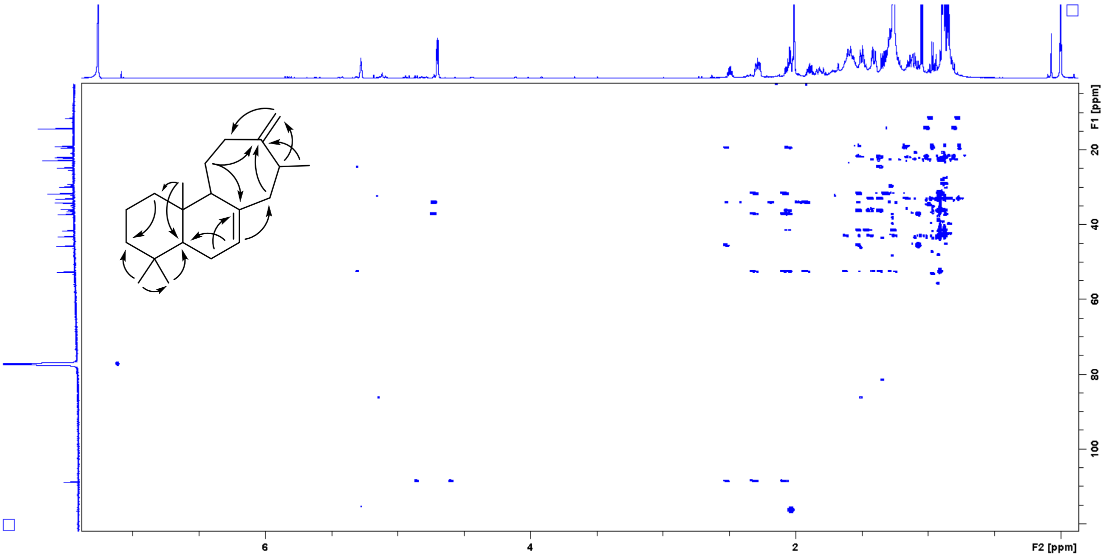

Figure S20. The NOESY spectrum of compound **2** in $CDCl_3$.

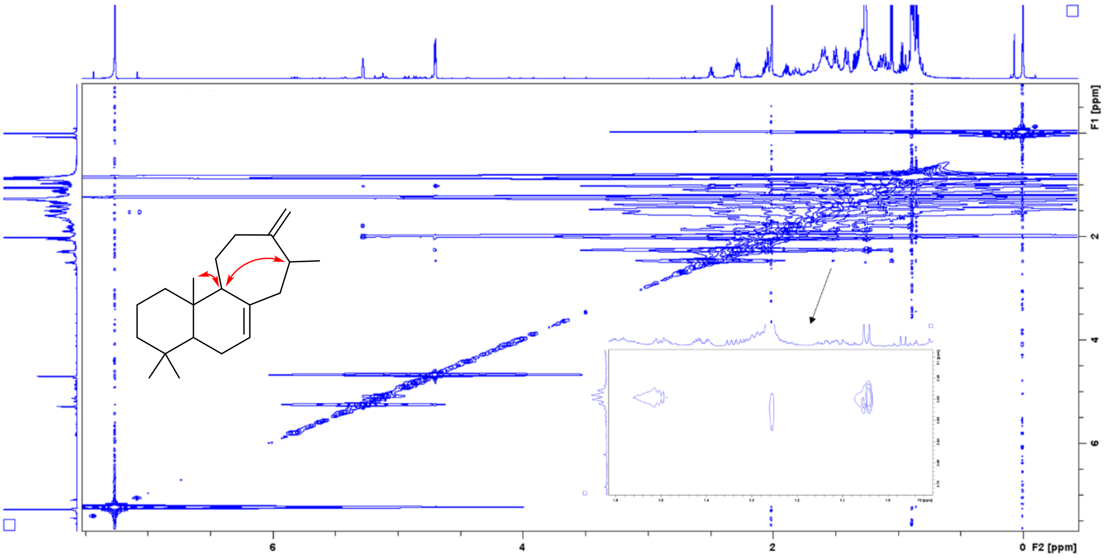

Figure S21. The DEPT 135° spectrum of compound **2** in $CDCl_3$.

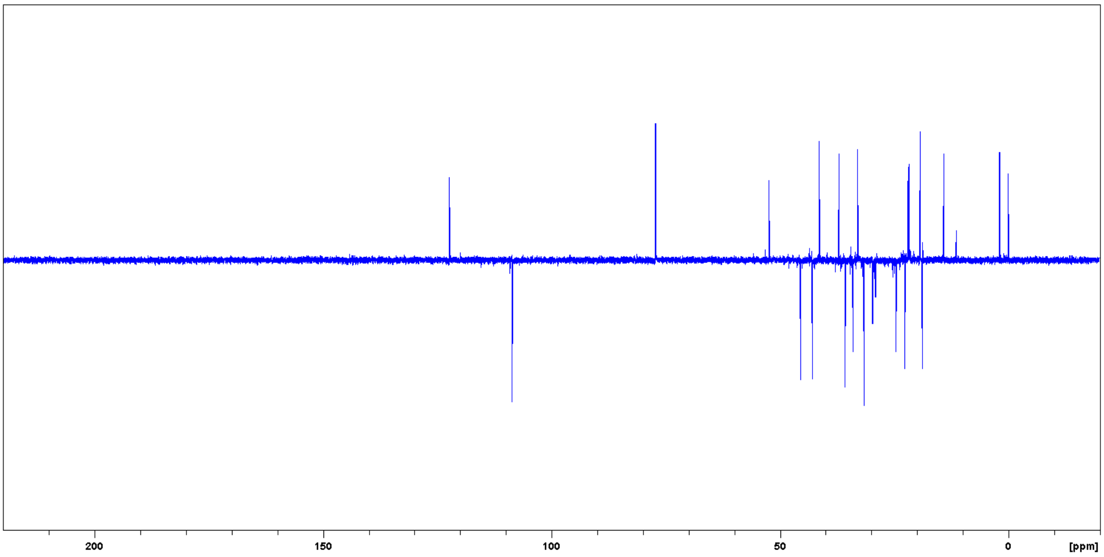

Figure S22. The IR spectrum of compound **2**.

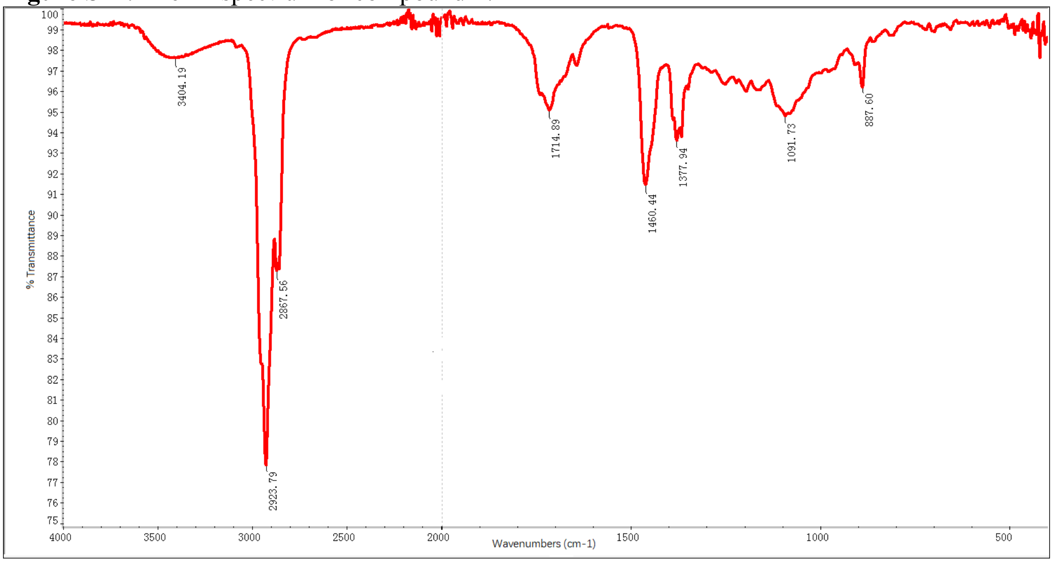

Figure S23. The HREIMS spectrum of compound **2**

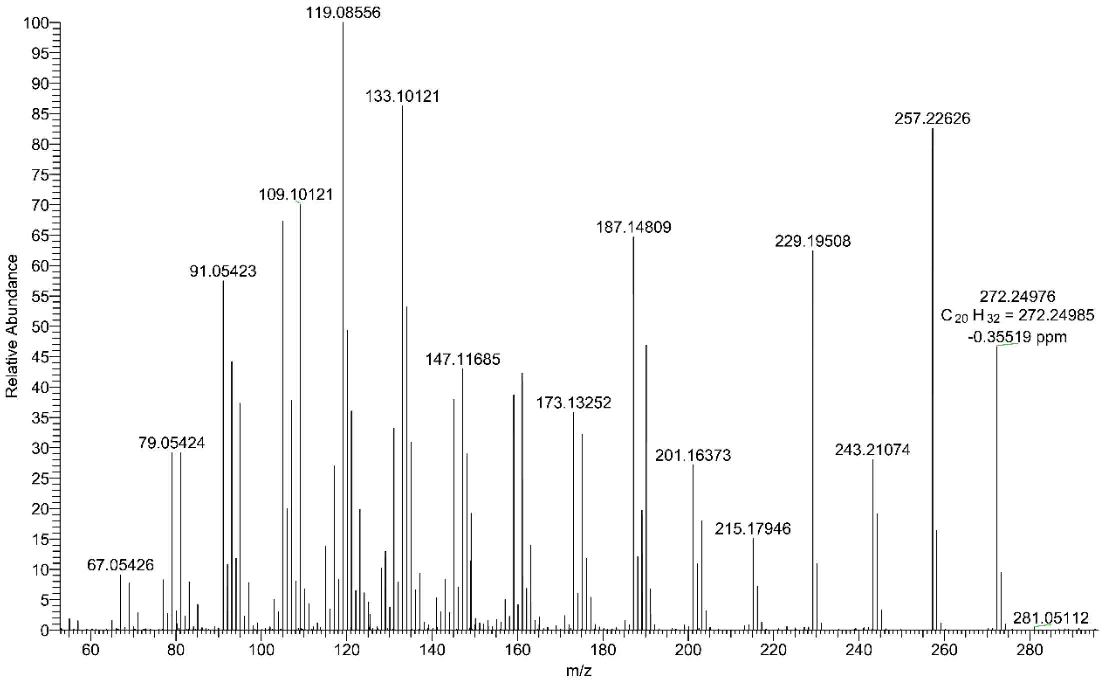

Figure S24. The ^{1}H NMR (600 MHz) and ^{13}C NMR (150 MHz) spectrum of compound **3** in $CDCl_3$.

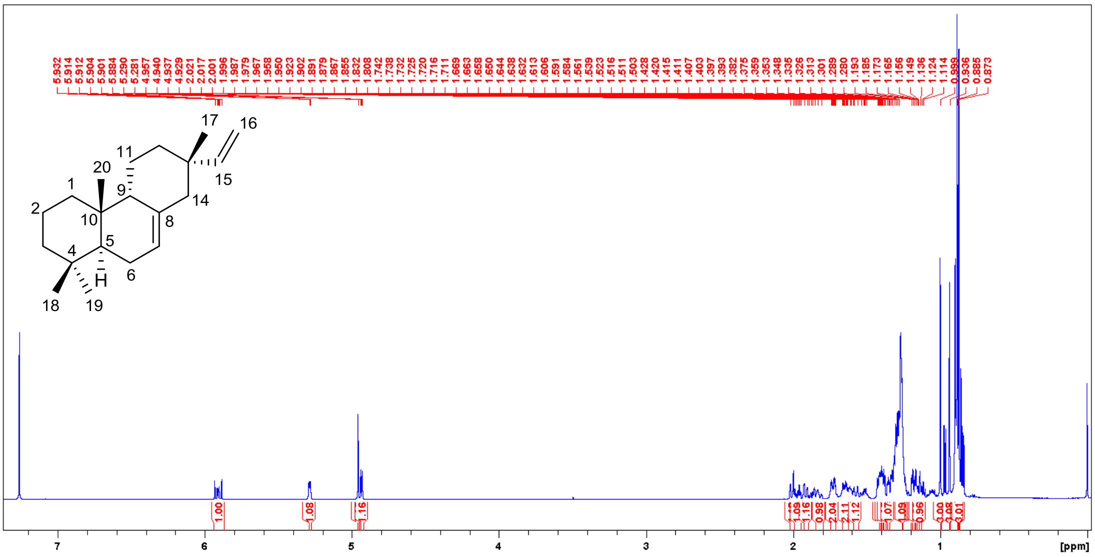

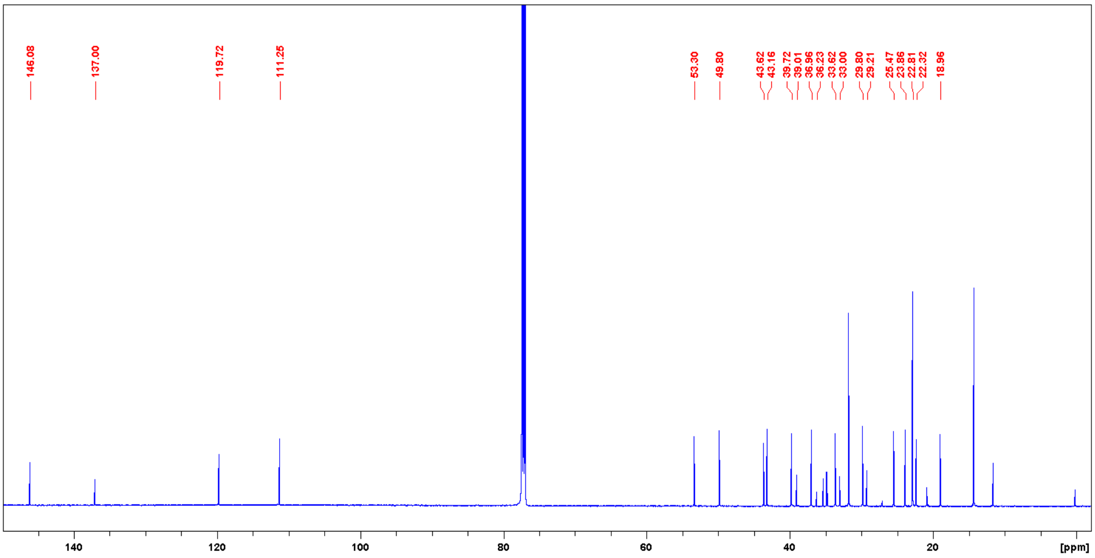

Figure S25. The ^{1}H NMR (600 MHz) and ^{13}C NMR (150 MHz) spectrum of compound **4** in $CDCl_3$.

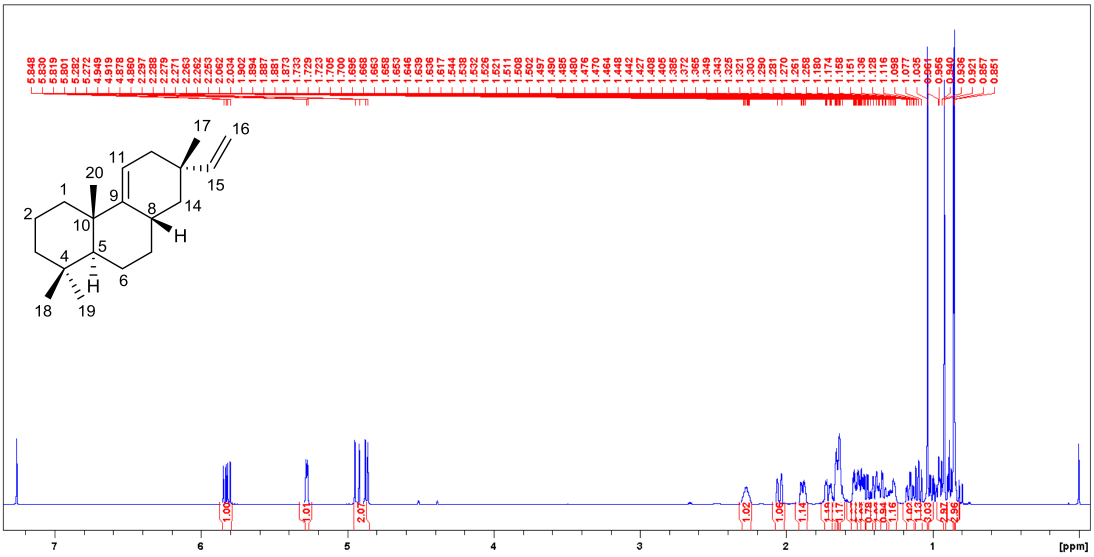

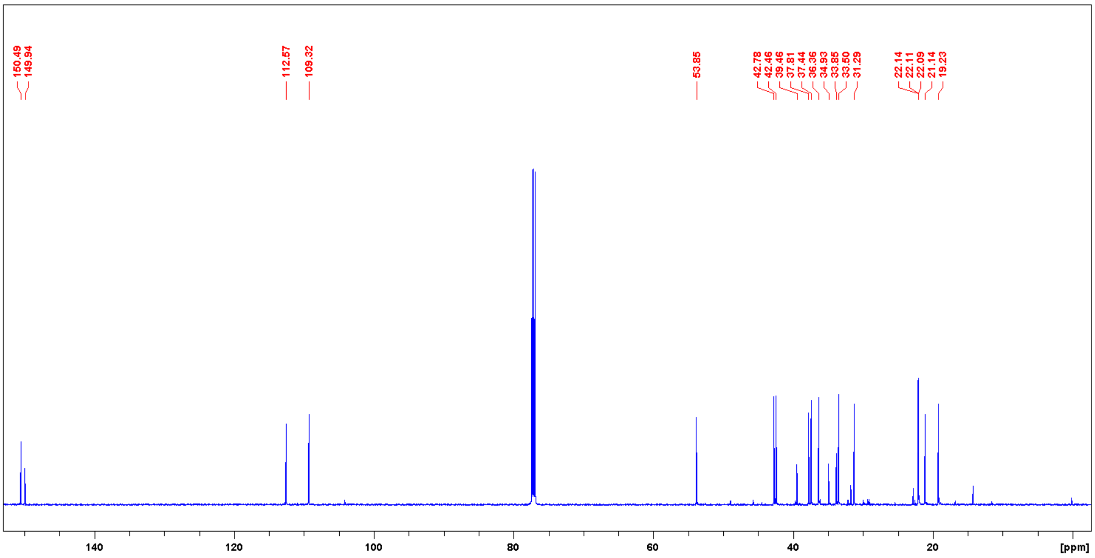

Figure S26. The ^{1}H NMR (600 MHz) and ^{13}C NMR (150 MHz) spectrum of compound **5** in $CDCl_3$.

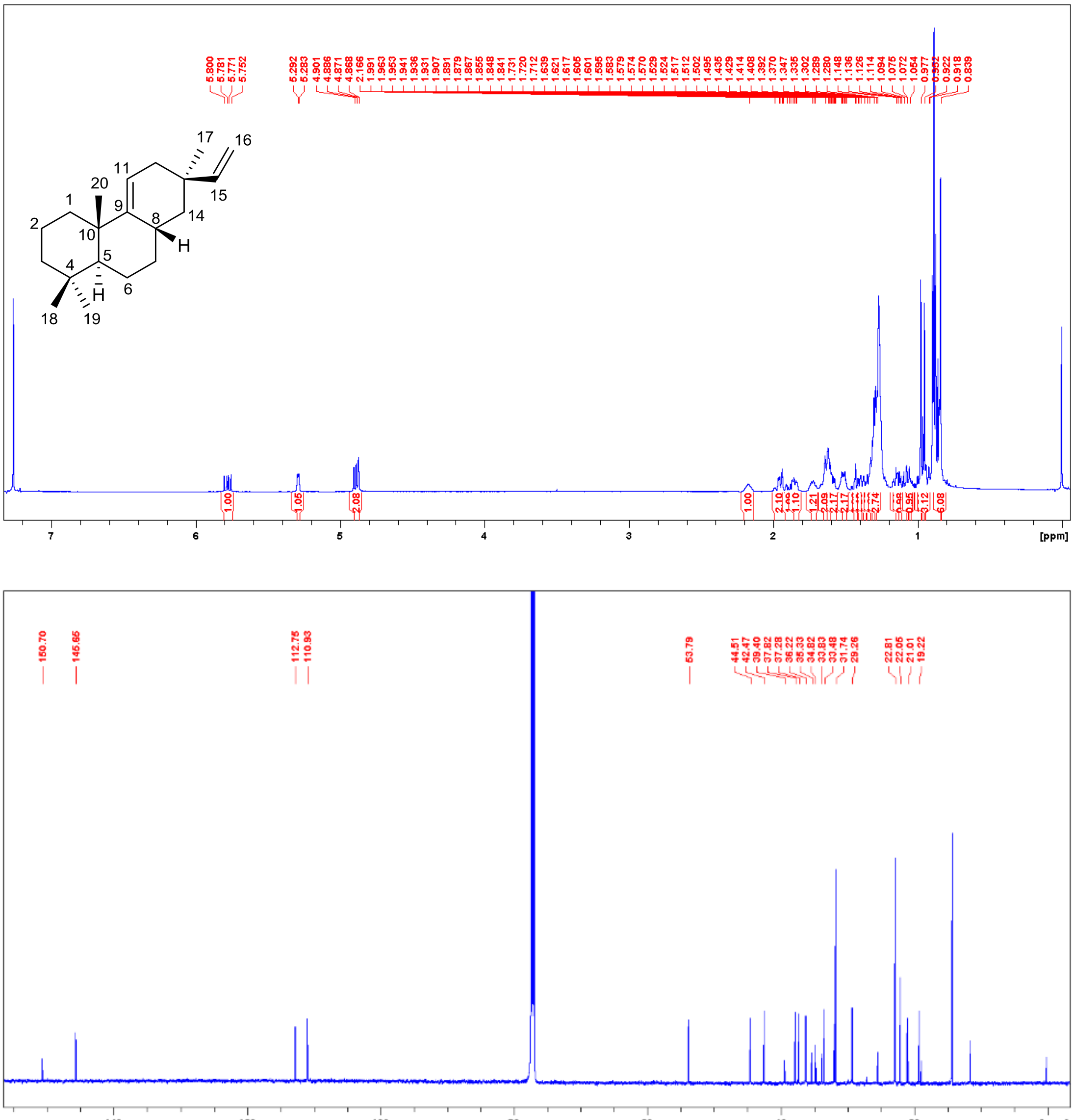

Figure S27. The ^{1}H NMR (600 MHz) and ^{13}C NMR (150 MHz) spectrum of compound **6** in $CDCl_3$.

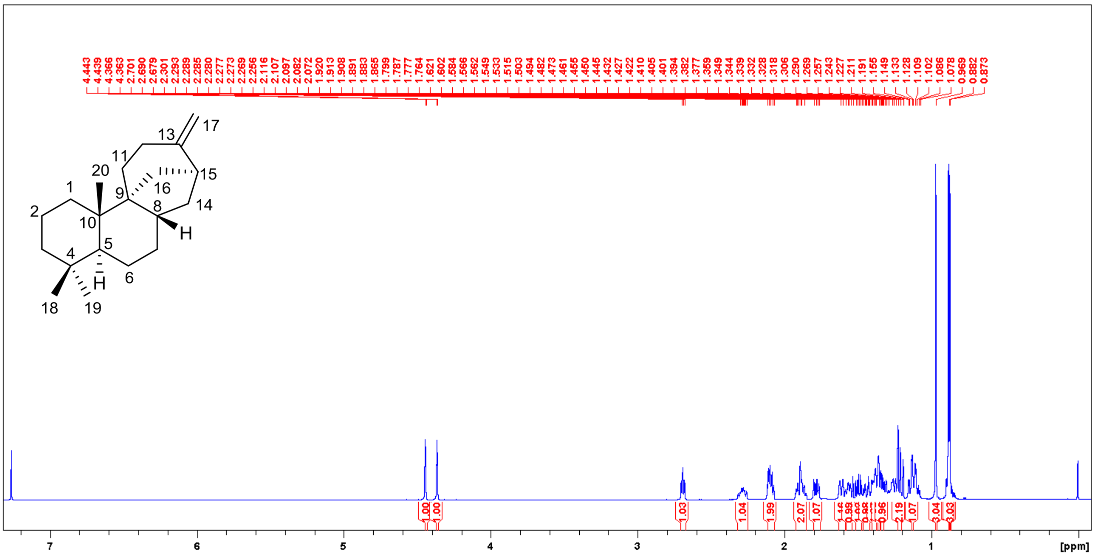

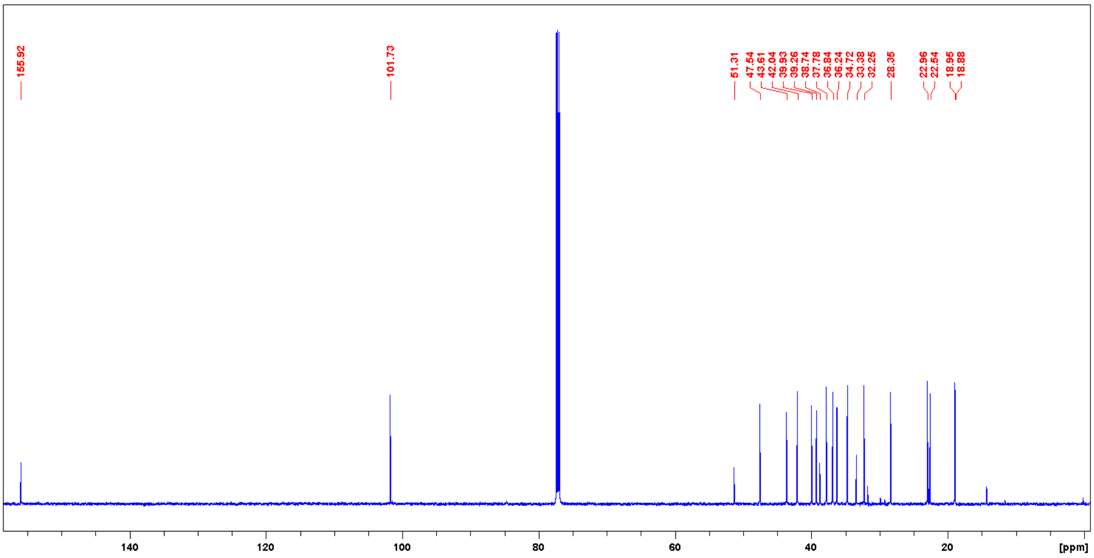

Figure S28. The ^{1}H NMR (600 MHz) and ^{13}C NMR (150 MHz) spectrum of compound **7** in $CDCl_3$.

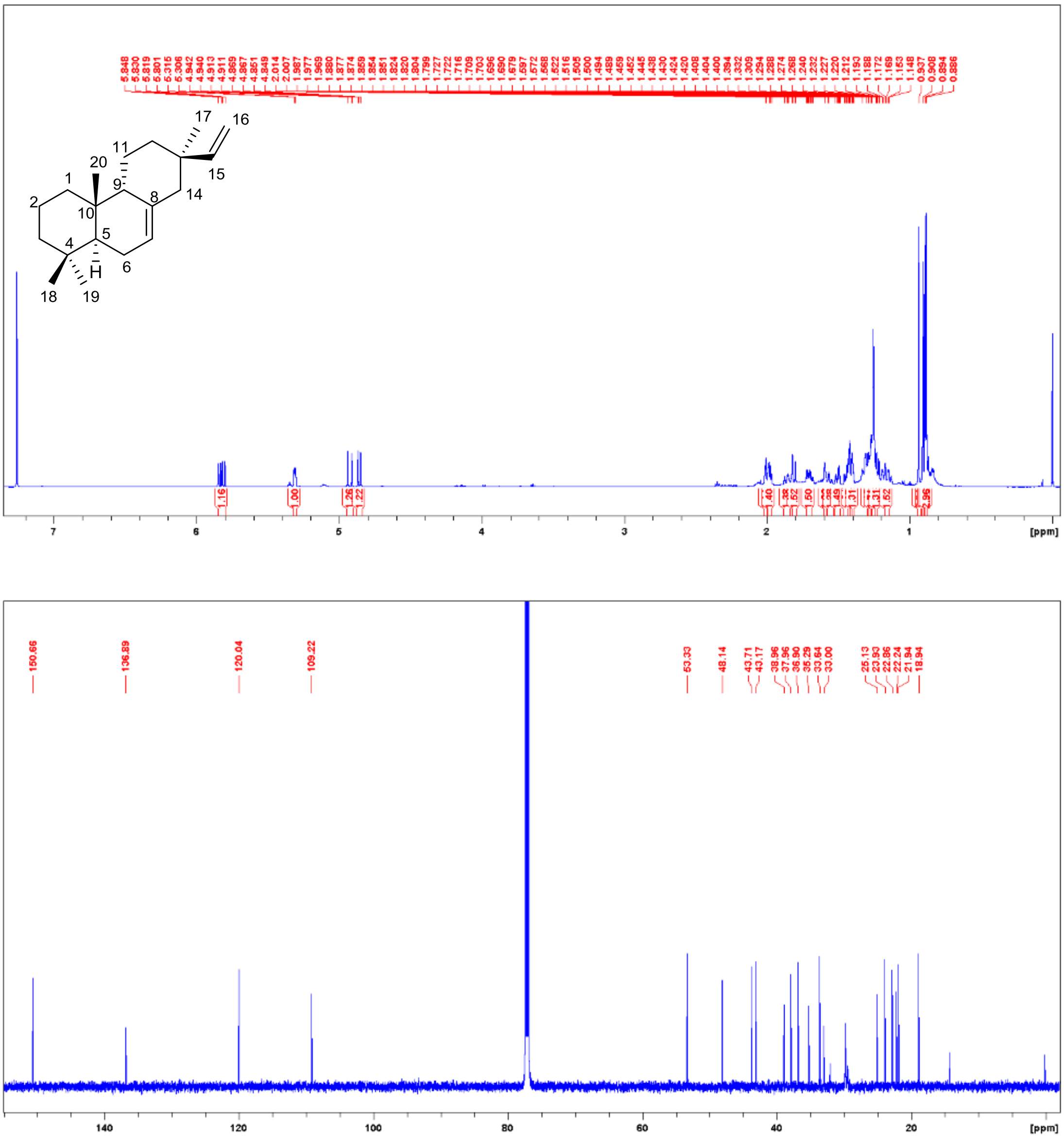

Figure S29. The ^{1}H NMR (600 MHz) and ^{13}C NMR (150 MHz) spectrum of compound **8** in $CDCl_3$.

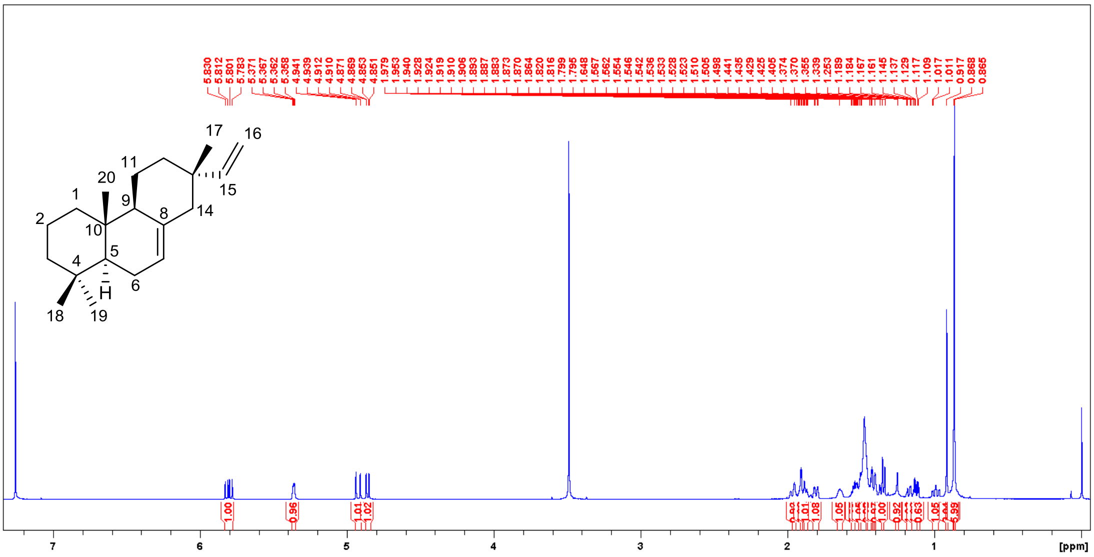

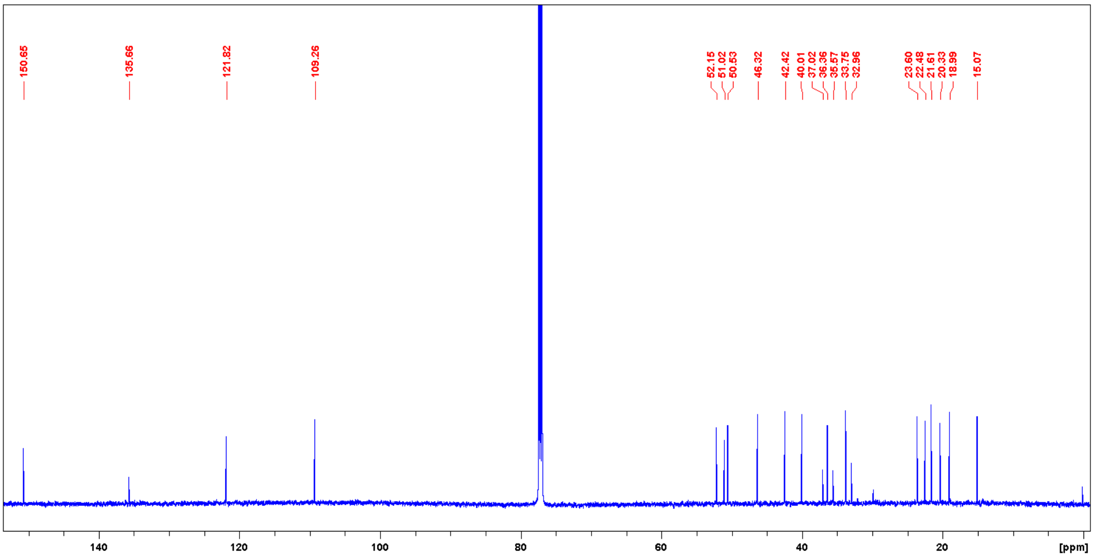

Figure S30. The ^{1}H NMR (600 MHz) and ^{13}C NMR (150 MHz) spectrum of compound **9** in $CDCl_3$.

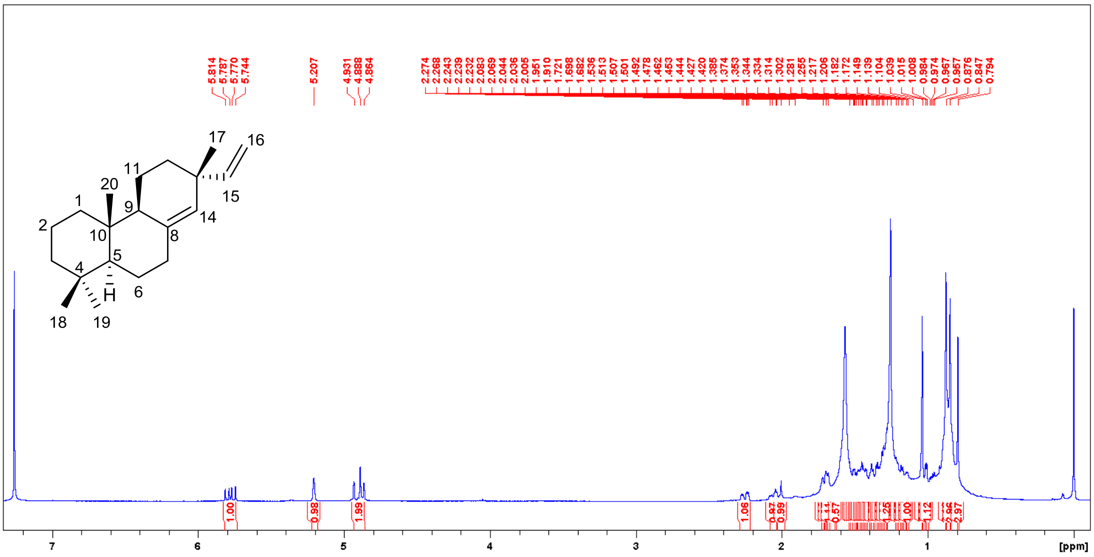

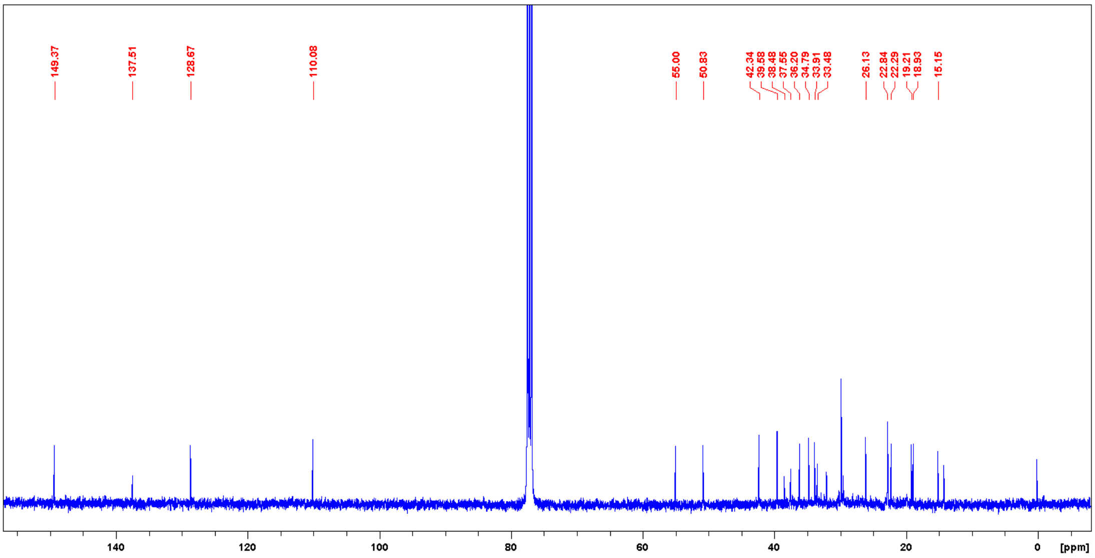

Supplementary References

1. Hindra *et al*, Strain prioritization for natural product discovery by a high-throughput real-time PCR method. *J. Nat. Prod.* **77**, 2296-2303 (2014).

2. Y. J. Zhou *et al*, Modular pathway engineering of diterpenoid synthases and the mevalonic acid pathway for miltiradiene production. *J. Am. Chem. Soc.* **134**, 3234-3241 (2012).

3. M. M. Xu, M. L. Hillwig, S. Prisic, R. M. Coates, R. J. Peters, Functional identification of rice *syn*-copalyl diphosphate synthase and its role in initiating biosynthesis of diterpenoid phytoalexin/allelopathic natural products. *Plant J.* **39**, 309-318 (2004).

4. C. Nakano, M. Oshima, N. Kurashima, T. Hoshino, Identification of a new diterpene biosynthetic gene cluster that produces O-methylkolavelool in *Herpetosiphon aurantiacus. Chembiochem* **16**, 772-781 (2015).

5. T. Dairi *et al*, Eubacterial diterpene cyclase genes essential for production of the isoprenoid antibiotic terpentecin. *J. Bacteriol.* **183**, 6085-6094 (2001).

6. F. M. Mann *et al*, Characterization and inhibition of a class II diterpene cyclase from *Mycobacterium tuberculosis*: implications for tuberculosis. *J. Biol. Chem.* **284**, 23574-23579 (2009).

7. M. M. Xu *et al*, Characterization of an orphan diterpenoid biosynthetic operon from *Salinispora arenicola. J. Nat. Prod.* **77**, 2144-2147 (2014).

8. M. M. Xu, M. L. Hillwig, M. S. Tiernan, R. J. Peters, Probing labdane-related diterpenoid biosynthesis in the fungal *Genus aspergillus*. *J. Nat. Prod.* **80**, 328-333 (2017).

9. M. R. Jia, S. K. Mishra, S. Tufts, R. L. Jernigan, R. J. Peters, Combinatorial biosynthesis and the basis for substrate promiscuity in class I diterpene synthases. *Metab. Eng.* **55**, 44-58 (2019).

10. H. Oikawa *et al*, Diversity of diterpene hydrocarbons in fungus *Phomabetae*. *Tetrahedron Lett.* **42**, 2329-2332 (2001).

11. D. Morrone *et al*, An unexpected diterpene cyclase from rice: functional identification of a stemodene synthase. *Arch. Biochem. Biophys.* **448**, 133-140 (2006).

12. Z. F. Ye *et al*, Biochemical synthesis of uniformly (13)C-labeled diterpene hydrocarbons and their bioconversion to diterpenoid phytoalexins in planta. *Biosci Biotechnol Biochem.* **81**, 1176-1184 (2017).

13. P. Tungcharoen *et al*, Anti-inflammatory effect of isopimarane diterpenoids from *Kaempferia galanga. Phytother. Res.* **34**, 612-623 (2020).

14. Z. F. Ye *et al*, Biochemical synthesis of uniformly (13)C-labeled diterpene hydrocarbons and their bioconversion to diterpenoid phytoalexins in planta. *Biosci. Biotechnol. Biochem.* **81**, 1176-1184 (2017).

15. E. Wenkert, B. L. Buckwalter, Cabon-13 nuclear magnetic resonance spectroscopy of naturally occurring substances. X. Pimaradienes. *J. Am. Chem. Soc.* **94**, 4367-4369 (1972).

16. L. Q. Zhang *et al*, Eutypenoids A-C: Novel Pimarane diterpenoids from the arctic fungus *eutypella* sp. D-1. *Mar. Drugs.* **14**, 44 (2016).

17. G. Gkinis *et al*, Parnapimarol and nepetaparnone from *Nepeta parnassica*. *J. Nat. Prod.* **71**, 926-928 (2008).

18. G. X. Na *et al*, Two pimarane diterpenoids from *Ephemerantha lonchophylla* and their evaluation as modulators of the multidrug resistance phenotype. *J. Nat. Prod.* **61**, 112-115 (1998).

19. X. T. Hou *et al*, Sarcosenones A-C, highly oxygenated pimarane diterpenoids from an endolichenic

fungus *Sarcosomataceae* sp.[†] *RSC Advances.* **10**, 15622-15628 (2020).

20. D. C. Oh, P. R. Jensen, C. A. Kauffman, W. Fenical, Libertellenones A-D: induction of cytotoxic diterpenoid biosynthesis by marine microbial competition. *Bioorg. Med. Chem.* **13**, 5267-5273 (2005).

21. X. D. Li *et al*, Aspewentins D-H, 20-*nor*-isopimarane derivatives from the deep sea sediment-derived fungus *Aspergillus wentii* SD-310. *J. Nat. Prod.* **79**, 1347-1353 (2016).

22. T. Masuda, K. Masuda, S. Shiragami, A. Jitoe, N. Nakatani, Orthosiphol A and B, novel diterpenoid inhibitors of TPA (12-O-tetradecanoylphorbol-13-acetate)-induced inflammation, from *Orthosiphon stamineus*. *Tetrahedron* **48**, 6787-6792 (1992).

23. S. Yoshida *et al*, ChemInform abstract: Four pimarane diterpenes from marine fungus: chloroform incorporated in crystal lattice for absolute configuration analysis by X-ray. *Cheminform* **39**, u0200 (2008).

24. V. Costantino *et al*, Tedanol: a potent anti-inflammatory *ent*-pimarane diterpene from the Caribbean Sponge *Tedania ignis*. *Bioorg. Med. Chem.* **17**, 7542-7547 (2009).

25. X. D. Wu, Diterpenoids from the twigs and leaves of *Fokienia hodginsii*. *J. Nat. Prod.* **76**, 1032-1038 (2013).

26. P. Reveglia *et al*, Pimarane diterpenes: Natural source, stereochemical configuration, and biological activity. *Chirality* **30**, 1115-1134 (2018).

27. S. M. Isyaka *et al*, *Ent*-abietane and *ent*-pimarane diterpenoids from *Croton mubango* (Euphorbiaceae). *Phytochemistry* **170**, 112217 (2020).

28. J. Kang, R. Y. Chen, D. Q. Yu, A new isopimarane-type diterpene and a new natural atisane-type diterpene from *Excoecaria agallocha*. *J. Asian. Nat. Prod. Res.* **7**, 729-734 (2005).

29. J. C. Li *et al*, Bioactive *ent*-isopimarane diterpenoids from *Euphorbia neriifolia*. *Phytochemistry* **175**, 112373 (2020).

30. W. J. Wei *et al*, Phytotoxic *ent*-isopimarane-type diterpenoids from *Euphorbia hylonoma*. *J. Nat. Prod.* **81**, 2381-2391 (2018).

31. M. Zhao, J. Cheng, B. Guo, J. Duan, C. T. Che, Momilactone and related diterpenoids as potential agricultural chemicals. *J. Agric. Food. Chem.* **66**, 7859-7872 (2018).

32. F. J. Schmitz, D. P. Michaud, P. G. Schmidt, Marine natural products: parguerol, deoxyparguerol, and isoparguerol. New brominated diterpenes with modified pimarane skeletons from the sea hare *Aplysia dactylomela*. *J. Am. Chem. Soc.* **104**, 6415-6423 (1982).

33. H. Lyu *et al*, Two 9,10-*syn*-pimarane diterpenes from the roots of *Lonicera macranthoides*. *Phytochemistry Lett.* **25**, 175-179 (2018).

34. E. Adou *et al*, Cytotoxic diterpenoids from two lianas from the Suriname rainforest. B*ioorg. Med. Chem.* **13**, 6009-6014 (2005).

35. S. Centeno-Leija *et al*, The structure of (*E*)-biformene synthase provides insights into the biosynthesis of bacterial bicyclic labdane-related diterpenoids. *J. Struct. Biol.* **207**, 29-39 (2019).

36. W. T. Liu *et al*, Structure, function and inhibition of *ent*-kaurene synthase from *Bradyrhizobium japonicum*. *Sci. Rep.* **4**, 6214 (2014).

37. R. Janke, C. Gorner, M. Hirte, T. Bruck, B. Loll, The first structure of a bacterial diterpene cyclase: CotB2. *Acta. Crystallogr. D. Biol. Crystallogr.* **70**, 1528-1537 (2014).

38. M. B. Chen, W. K. Chou, T. Toyomasu, D. E. Cane, D. W. Christianson, Structure and function of

fusicoccadiene synthase, a hexameric bifunctional diterpene synthase. *ACS Chem. Biol.* **11**, 889-899 (2016).

39. H. B. He *et al*, Discovery of the cryptic function of terpene cyclases as aromatic prenyltransferases. *Nat. Commun.* **11**, 3958 (2020).

40. K. Schriever *et al*, Engineering of ancestors as a tool to elucidate structure, mechanism, and specificity of extant terpene cyclase. *J. Am. Chem. Soc.*, in press.

41. H. C. Chan *et al*, Structure and inhibition of tuberculosinol synthase and decaprenyl diphosphate synthase from *Mycobacterium tuberculosis*. *J. Am. Chem. Soc.* **136**, 2892-2896 (2014).

42. M. Koksal, Y. H. Jin, R. M. Coates, R. Croteau, D. W. Christianson, Taxadiene synthase structure and evolution of modular architecture in terpene biosynthesis. *Nature* **469**, 116-120 (2011).

43. J. D. Rudolf *et al*, Structure of the *ent*-copalyl diphosphate synthase PtmT2 from *Streptomyces platensis* CB00739, a bacterial type II diterpene synthase. *J. Am. Chem. Soc.* **138**, 10905-10915 (2016).

44. M. Koksal, H. Y. Hu, R. M. Coates, R. J. Peters, D. W. Christianson, Structure and mechanism of the diterpene cyclase *ent*-copalyl diphosphate synthase. *Nat. Chem. Biol.* **7**, 431-433 (2011).

45. K. Zhou *et al*, Insights into diterpene cyclization from structure of bifunctional abietadiene synthase from *Abies grandis*. *J. Biol. Chem.* **287**, 6840-6850 (2012).
