# Supplementary material for: Functional characterization and structural bases of two class I diterpene synthases in pimarane-type diterpene biosynthesis
Source: Commun Chem. 2021 Sep 30;4:140. doi: 10.1038/s42004-021-00578-z (PMC9814573; doi:10.1038/s42004-021-00578-z)
Supplement: Supplementary file 3 — Supplementary Data-1 [file 42004_2021_578_MOESM3_ESM.pdf]

Full wwPDB X-ray Structure Validation Report (i)

Feb 17, 2021 – 05:20 PM JST

PDB ID	:	7E4N
Title	:	Sat1646
Deposited on	:	2021-02-14
Resolution	:	1.94 Å(reported)

This is a Full wwPDB X-ray Structure Validation Report.

This report is produced by the wwPDB biocuration pipeline after annotation of the structure.

We welcome your comments at *validation@mail.wwpdb.org*
A user guide is available at
https://www.wwpdb.org/validation/2017/XrayValidationReportHelp
with specific help available everywhere you see the (i) symbol.

The following versions of software and data (see references (i)) were used in the production of this report:

MolProbity	:	4.02b-467
Xtriage (Phenix)	:	1.13
EDS	:	2.16
buster-report	:	1.1.7 (2018)
Percentile statistics	:	20191225.v01 (using entries in the PDB archive December 25th 2019)
Refmac	:	5.8.0158
CCP4	:	7.0.044 (Gargrove)
Ideal geometry (proteins)	:	Engh & Huber (2001)
Ideal geometry (DNA, RNA)	:	Parkinson et al. (1996)
Validation Pipeline (wwPDB-VP)	:	2.16

CONFIDENTIAL VALIDATION REPORT

1 Overall quality at a glance (i)

The following experimental techniques were used to determine the structure:
X-RAY DIFFRACTION

The reported resolution of this entry is 1.94 Å.

Percentile scores (ranging between 0-100) for global validation metrics of the entry are shown in the following graphic. The table shows the number of entries on which the scores are based.

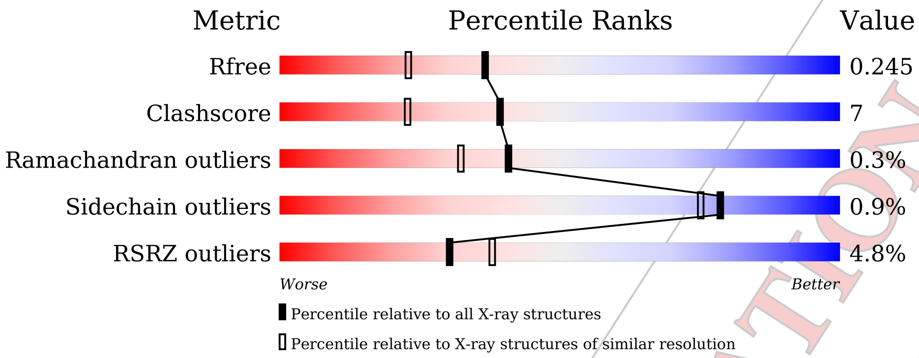

Metric	Whole archive (#Entries)	Similar resolution (#Entries, resolution range(Å))
R_{free}	130704	4310 (1.96-1.92)
Clashscore	141614	1023 (1.94-1.94)
Ramachandran outliers	138981	1007 (1.94-1.94)
Sidechain outliers	138945	1007 (1.94-1.94)
RSRZ outliers	127900	4250 (1.96-1.92)

The table below summarises the geometric issues observed across the polymeric chains and their fit to the electron density. The red, orange, yellow and green segments of the lower bar indicate the fraction of residues that contain outliers for >=3, 2, 1 and 0 types of geometric quality criteria respectively. A grey segment represents the fraction of residues that are not modelled. The numeric value for each fraction is indicated below the corresponding segment, with a dot representing fractions <=5% The upper red bar (where present) indicates the fraction of residues that have poor fit to the electron density. The numeric value is given above the bar.

Mol	Chain	Length	Quality of chain
1	A	299	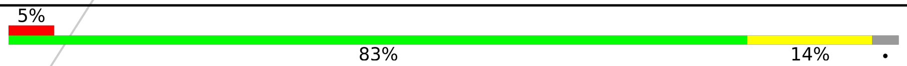

CONFIDENTIAL VALIDATION REPORT

2 Entry composition (i)

There are 3 unique types of molecules in this entry. The entry contains 2409 atoms, of which 0 are hydrogens and 0 are deuteriums.

In the tables below, the ZeroOcc column contains the number of atoms modelled with zero occupancy, the AltConf column contains the number of residues with at least one atom in alternate conformation and the Trace column contains the number of residues modelled with at most 2 atoms.

- Molecule 1 is a protein called Sat1646.

Mol	Chain	Residues	Atoms					ZeroOcc	AltConf	Trace
			Total	C	N	O	S			
1	A	290	2267	1406	422	429	10	0	0	0

- Molecule 2 is MAGNESIUM ION (three-letter code: MG) (formula: Mg) (labeled as "Ligand of Interest" by depositor).

Mol	Chain	Residues	Atoms		ZeroOcc	AltConf
			Total	Mg		
2	A	3	3	3	0	0

- Molecule 3 is water.

Mol	Chain	Residues	Atoms		ZeroOcc	AltConf
			Total	O		
3	A	139	139	139	0	0

CONFIDENTIAL VALIDATION REPORT

3 Residue-property plots ⓘ

These plots are drawn for all protein, RNA, DNA and oligosaccharide chains in the entry. The first graphic for a chain summarises the proportions of the various outlier classes displayed in the second graphic. The second graphic shows the sequence view annotated by issues in geometry and electron density. Residues are color-coded according to the number of geometric quality criteria for which they contain at least one outlier: green = 0, yellow = 1, orange = 2 and red = 3 or more. A red dot above a residue indicates a poor fit to the electron density (RSRZ > 2). Stretches of 2 or more consecutive residues without any outlier are shown as a green connector. Residues present in the sample, but not in the model, are shown in grey.

- Molecule 1: Sat1646

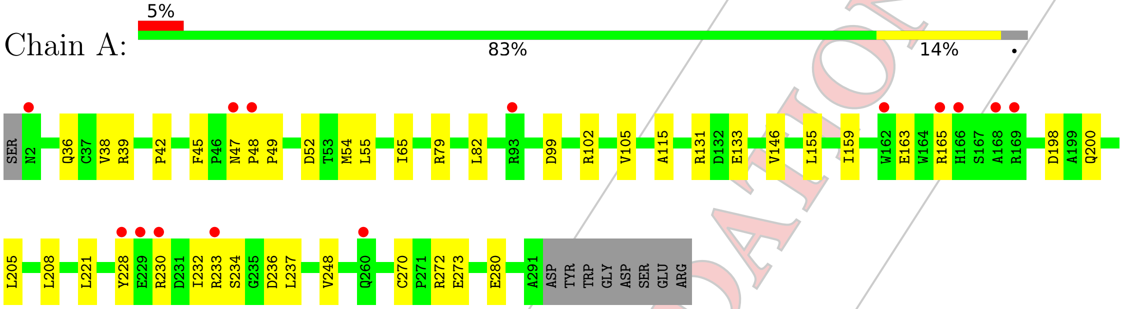

CONFIDENTIAL VALIDATION REPORT

4 Data and refinement statistics (i)

Property	Value	Source
Space group	P 42 21 2	Depositor
Cell constants<br>a, b, c, α, β, γ	89.02Å 89.02Å 68.67Å<br>90.00° 90.00° 90.00°	Depositor
Resolution (Å)	28.61 – 1.94<br>62.94 – 1.94	Depositor<br>EDS
% Data completeness<br>(in resolution range)	86.3 (28.61-1.94)<br>86.4 (62.94-1.94)	Depositor<br>EDS
R_{merge}	0.11	Depositor
R_{sym}	(Not available)	Depositor
$< I/\sigma(I) >$ [1]	2.06 (at 1.94Å)	Xtriage
Refinement program	PHENIX 1.17.1_3660	Depositor
R, R_{free}	0.195 , 0.247<br>0.200 , 0.245	Depositor<br>DCC
R_{free} test set	936 reflections (5.17%)	wwPDB-VP
Wilson B-factor (Å^2)	34.8	Xtriage
Anisotropy	0.093	Xtriage
Bulk solvent k_{sol}(e/Å^3), B_{sol}(Å^2)	0.37 , 44.8	EDS
L-test for twinning[2]	$< \|L\| > = 0.51$, $< L^2 > = 0.34$	Xtriage
Estimated twinning fraction	No twinning to report.	Xtriage
F_o,F_c correlation	0.95	EDS
Total number of atoms	2409	wwPDB-VP
Average B, all atoms (Å^2)	39.0	wwPDB-VP

Xtriage's analysis on translational NCS is as follows: *The largest off-origin peak in the Patterson function is 6.34% of the height of the origin peak. No significant pseudotranslation is detected.*

[1] Intensities estimated from amplitudes.

[2] Theoretical values of $< |L| >$, $< L^2 >$ for acentric reflections are 0.5, 0.333 respectively for untwinned datasets, and 0.375, 0.2 for perfectly twinned datasets.

CONFIDENTIAL VALIDATION REPORT

5 Model quality (i)

5.1 Standard geometry (i)

Bond lengths and bond angles in the following residue types are not validated in this section: MG

The Z score for a bond length (or angle) is the number of standard deviations the observed value is removed from the expected value. A bond length (or angle) with $|Z| > 5$ is considered an outlier worth inspection. RMSZ is the root-mean-square of all Z scores of the bond lengths (or angles).

Mol	Chain	Bond lengths		Bond angles	
		RMSZ	#|Z| >5	RMSZ	#|Z| >5
1	A	0.46	0/2316	0.55	0/3156

There are no bond length outliers.

There are no bond angle outliers.

There are no chirality outliers.

There are no planarity outliers.

5.2 Too-close contacts (i)

In the following table, the Non-H and H(model) columns list the number of non-hydrogen atoms and hydrogen atoms in the chain respectively. The H(added) column lists the number of hydrogen atoms added and optimized by MolProbity. The Clashes column lists the number of clashes within the asymmetric unit, whereas Symm-Clashes lists symmetry-related clashes.

Mol	Chain	Non-H	H(model)	H(added)	Clashes	Symm-Clashes
1	A	2267	0	2182	33	0
2	A	3	0	0	0	0
3	A	139	0	0	1	0
All	All	2409	0	2182	33	0

The all-atom clashscore is defined as the number of clashes found per 1000 atoms (including hydrogen atoms). The all-atom clashscore for this structure is 7.

All (33) close contacts within the same asymmetric unit are listed below, sorted by their clash magnitude.

Atom-1	Atom-2	Interatomic distance (Å)	Clash overlap (Å)
1:A:270:CYS:HB3	1:A:273:GLU:HB2	1.55	0.88

Continued on next page...

CONFIDENTIAL VALIDATION REPORT

Continued from previous page...

Atom-1	Atom-2	Interatomic distance (Å)	Clash overlap (Å)
1:A:208:LEU:CD1	1:A:273:GLU:HG2	2.14	0.77
1:A:208:LEU:HD11	1:A:273:GLU:HG2	1.75	0.67
1:A:79:ARG:HH21	1:A:133:GLU:HG3	1.62	0.63
1:A:208:LEU:HD22	1:A:273:GLU:HB3	1.82	0.60
1:A:208:LEU:HD13	1:A:273:GLU:HG2	1.83	0.59
1:A:105:VAL:HG13	1:A:155:LEU:HB3	1.87	0.57
1:A:165:ARG:HE	1:A:236:ASP:HA	1.70	0.56
1:A:208:LEU:CD2	1:A:273:GLU:HB3	2.36	0.56
1:A:39:ARG:O	1:A:42:PRO:HD3	2.06	0.56
1:A:36:GLN:OE1	1:A:39:ARG:NH2	2.41	0.54
1:A:272:ARG:NH1	3:A:409:HOH:O	2.42	0.53
1:A:228:TYR:CZ	1:A:232:ILE:HD11	2.44	0.53
1:A:165:ARG:NE	1:A:236:ASP:HA	2.24	0.52
1:A:38:VAL:HA	1:A:45:PHE:CD2	2.45	0.51
1:A:65:ILE:HG12	1:A:280:GLU:HA	1.92	0.51
1:A:102:ARG:HG2	1:A:102:ARG:HH11	1.77	0.49
1:A:115:ALA:O	1:A:131:ARG:NH2	2.46	0.48
1:A:165:ARG:HH21	1:A:237:LEU:H	1.61	0.48
1:A:198:ASP:OD1	1:A:200:GLN:HB2	2.13	0.48
1:A:79:ARG:NH2	1:A:133:GLU:HG3	2.28	0.47
1:A:47:ASN:HB3	1:A:48:PRO:HD3	1.97	0.46
1:A:237:LEU:HD12	1:A:237:LEU:HA	1.77	0.46
1:A:234:SER:OG	1:A:234:SER:O	2.30	0.44
1:A:105:VAL:HG21	1:A:159:ILE:HD11	2.00	0.43
1:A:230:ARG:HB3	1:A:233:ARG:NH1	2.33	0.43
1:A:146:VAL:HG11	1:A:205:LEU:HD13	2.01	0.43
1:A:102:ARG:NH2	1:A:163:GLU:OE2	2.52	0.42
1:A:230:ARG:O	1:A:233:ARG:HB3	2.20	0.42
1:A:165:ARG:NH2	1:A:236:ASP:HB3	2.36	0.41
1:A:221:LEU:HD22	1:A:248:VAL:HG13	2.03	0.40
1:A:82:LEU:HD23	1:A:82:LEU:HA	1.95	0.40
1:A:54:MET:HE2	1:A:55:LEU:HD23	2.04	0.40

There are no symmetry-related clashes.

5.3 Torsion angles (i)

5.3.1 Protein backbone (i)

In the following table, the Percentiles column shows the percent Ramachandran outliers of the chain as a percentile score with respect to all X-ray entries followed by that with respect to entries

CONFIDENTIAL VALIDATION REPORT

of similar resolution.

The Analysed column shows the number of residues for which the backbone conformation was analysed, and the total number of residues.

Mol	Chain	Analysed	Favoured	Allowed	Outliers	Percentiles	
1	A	288/299 (96%)	286 (99%)	1 (0%)	1 (0%)	41	32

All (1) Ramachandran outliers are listed below:

Mol	Chain	Res	Type
1	A	49	PRO

5.3.2 Protein sidechains ⓘ

In the following table, the Percentiles column shows the percent sidechain outliers of the chain as a percentile score with respect to all X-ray entries followed by that with respect to entries of similar resolution.

The Analysed column shows the number of residues for which the sidechain conformation was analysed, and the total number of residues.

Mol	Chain	Analysed	Rotameric	Outliers	Percentiles	
1	A	233/241 (97%)	231 (99%)	2 (1%)	78	75

All (2) residues with a non-rotameric sidechain are listed below:

Mol	Chain	Res	Type
1	A	52	ASP
1	A	99	ASP

Sometimes sidechains can be flipped to improve hydrogen bonding and reduce clashes. All (2) such sidechains are listed below:

Mol	Chain	Res	Type
1	A	166	HIS
1	A	260	GLN

5.3.3 RNA ⓘ

There are no RNA molecules in this entry.

CONFIDENTIAL VALIDATION REPORT

5.4 Non-standard residues in protein, DNA, RNA chains (i)

There are no non-standard protein/DNA/RNA residues in this entry.

5.5 Carbohydrates (i)

There are no monosaccharides in this entry.

5.6 Ligand geometry (i)

Of 3 ligands modelled in this entry, 3 are monoatomic - leaving 0 for Mogul analysis.

There are no bond length outliers.

There are no bond angle outliers.

There are no chirality outliers.

There are no torsion outliers.

There are no ring outliers.

No monomer is involved in short contacts.

5.7 Other polymers (i)

There are no such residues in this entry.

5.8 Polymer linkage issues (i)

There are no chain breaks in this entry.

CONFIDENTIAL VALIDATION REPORT

6 Fit of model and data (i)

6.1 Protein, DNA and RNA chains (i)

In the following table, the column labelled '#RSRZ> 2' contains the number (and percentage) of RSRZ outliers, followed by percent RSRZ outliers for the chain as percentile scores relative to all X-ray entries and entries of similar resolution. The OWAB column contains the minimum, median, 95th percentile and maximum values of the occupancy-weighted average B-factor per residue. The column labelled 'Q< 0.9' lists the number of (and percentage) of residues with an average occupancy less than 0.9.

Mol	Chain	Analysed	<RSRZ>	#RSRZ>2			OWAB(Å^2)	Q<0.9
1	A	290/299 (96%)	0.38	14 (4%)	30	38	23, 35, 63, 90	0

All (14) RSRZ outliers are listed below:

Mol	Chain	Res	Type	RSRZ
1	A	47	ASN	4.1
1	A	168	ALA	4.0
1	A	230	ARG	3.9
1	A	2	ASN	3.4
1	A	166	HIS	3.4
1	A	169	ARG	3.1
1	A	48	PRO	3.0
1	A	165	ARG	2.9
1	A	93	ARG	2.7
1	A	229	GLU	2.6
1	A	233	ARG	2.3
1	A	260	GLN	2.2
1	A	162	TRP	2.1
1	A	228	TYR	2.0

6.2 Non-standard residues in protein, DNA, RNA chains (i)

There are no non-standard protein/DNA/RNA residues in this entry.

6.3 Carbohydrates (i)

There are no monosaccharides in this entry.

CONFIDENTIAL VALIDATION REPORT

6.4 Ligands (i)

In the following table, the Atoms column lists the number of modelled atoms in the group and the number defined in the chemical component dictionary. The B-factors column lists the minimum, median, 95th percentile and maximum values of B factors of atoms in the group. The column labelled 'Q< 0.9' lists the number of atoms with occupancy less than 0.9.

Mol	Type	Chain	Res	Atoms	RSCC	RSR	B-factors(Å^2)	Q<0.9
2	MG	A	302	1/1	0.84	0.20	59,59,59,59	0
2	MG	A	301	1/1	0.89	0.30	50,50,50,50	0
2	MG	A	303	1/1	0.95	0.08	64,64,64,64	0

The following is a graphical depiction of the model fit to experimental electron density of all instances of the Ligand of Interest. In addition, ligands with molecular weight > 250 and outliers as shown on the geometry validation Tables will also be included. Each fit is shown from different orientation to approximate a three-dimensional view.

CONFIDENTIAL VALIDATION REPORT

Electron density around MG A 302:

2mF$_o$-DF$_c$ (at 0.7 rmsd) in gray
mF$_o$-DF$_c$ (at 3 rmsd) in purple (negative)
and green (positive)

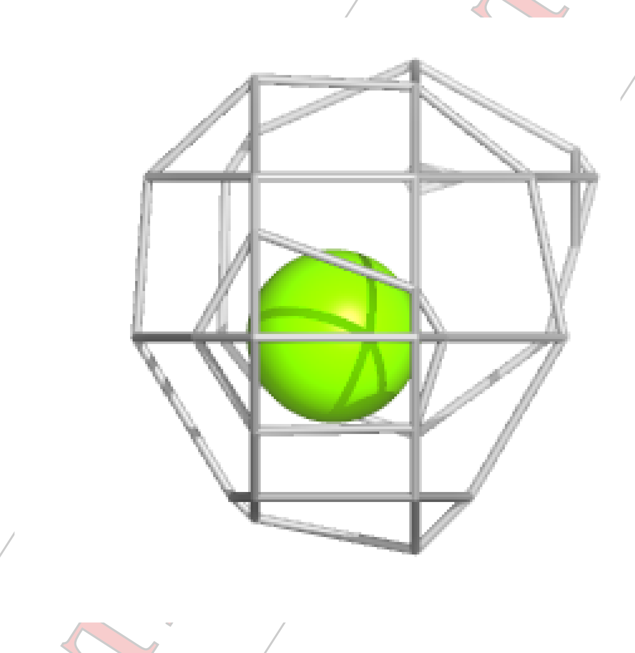

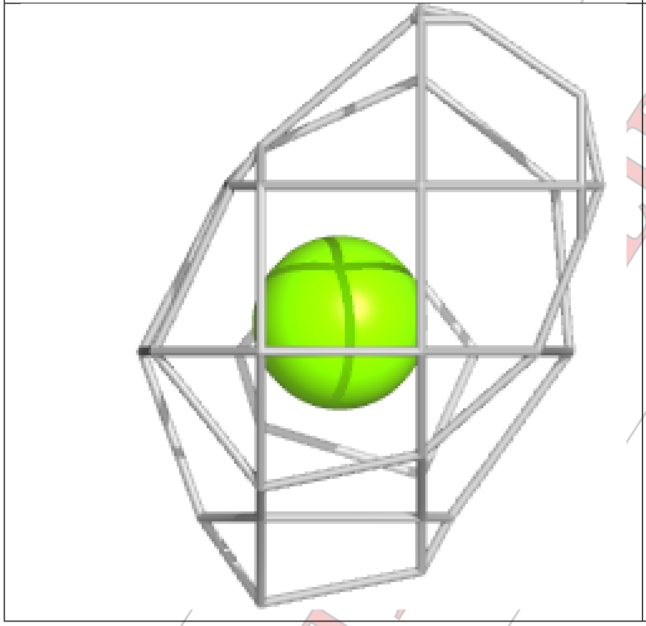

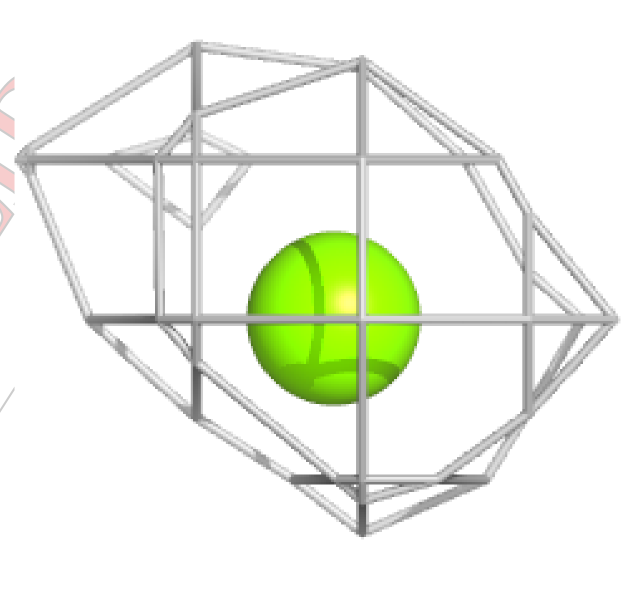

CONFIDENTIAL

Electron density around MG A 301:

2mF$_o$-DF$_c$ (at 0.7 rmsd) in gray
mF$_o$-DF$_c$ (at 3 rmsd) in purple (negative)
and green (positive)

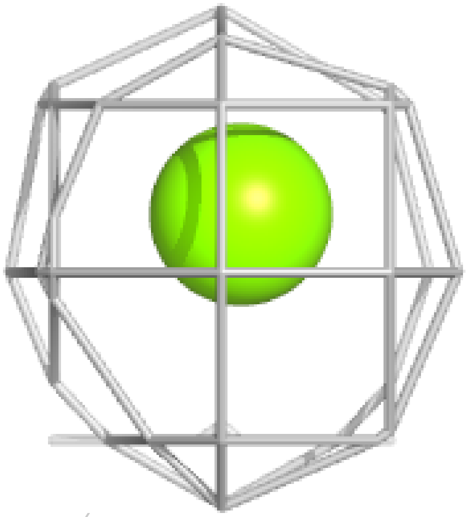

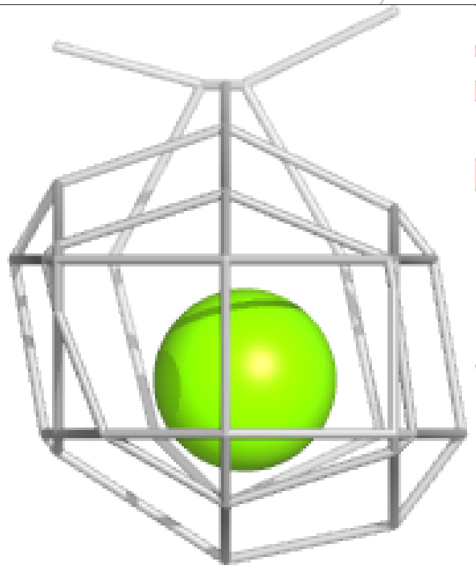

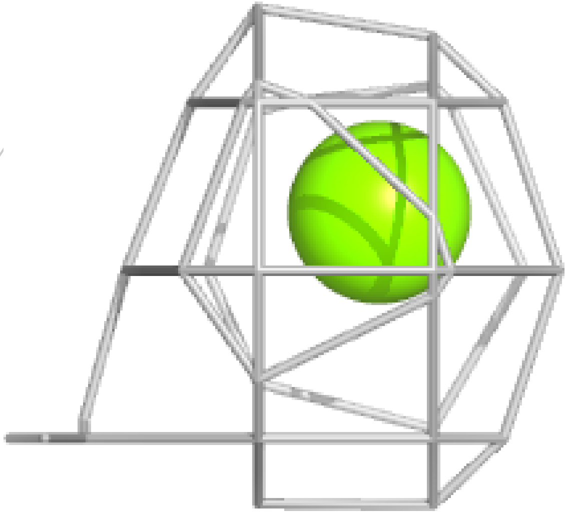

Electron density around MG A 303:

$2mF_o$-DF_c (at 0.7 rmsd) in gray
mF_o-DF_c (at 3 rmsd) in purple (negative)
and green (positive)

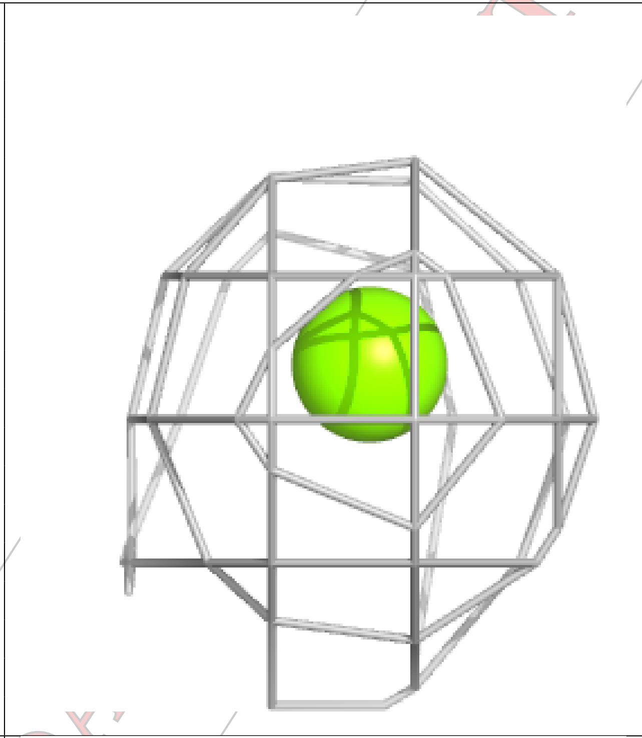

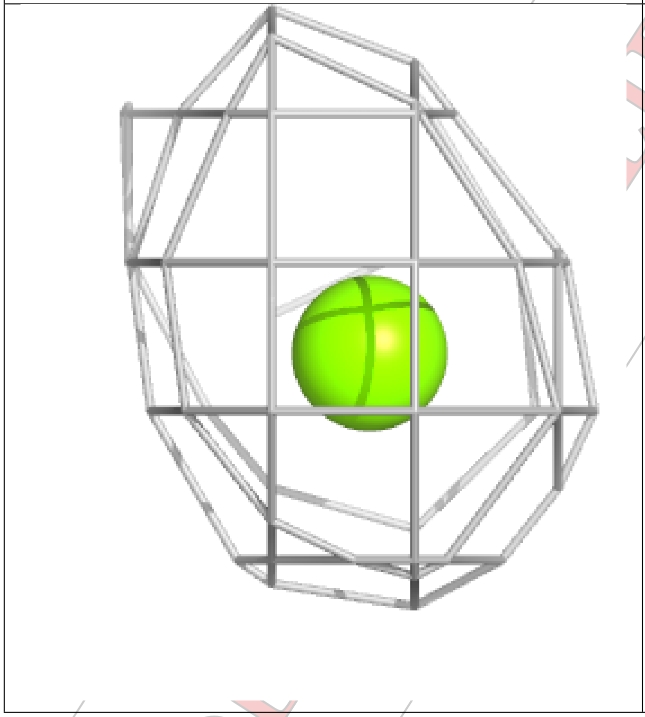

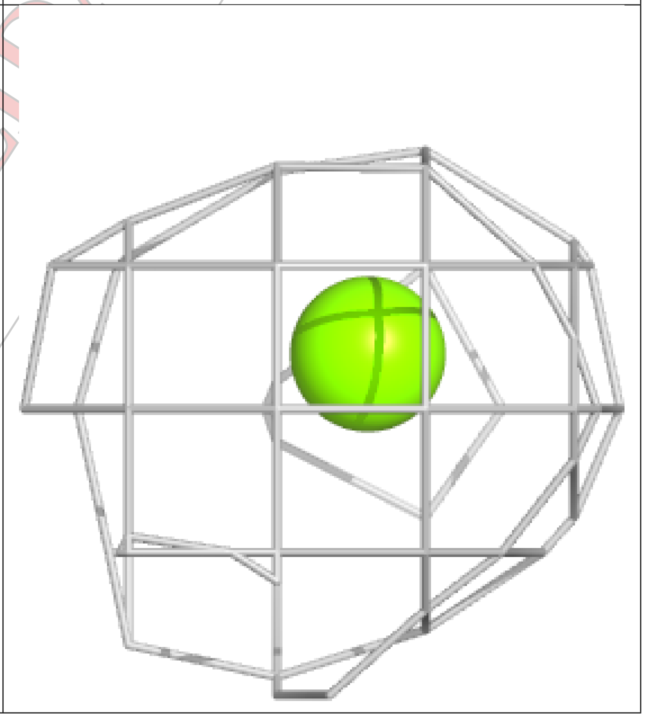

6.5 Other polymers ⓘ

There are no such residues in this entry.
