# Supplementary material for: Functional characterization and structural bases of two class I diterpene synthases in pimarane-type diterpene biosynthesis
Source: Commun Chem. 2021 Sep 30;4:140. doi: 10.1038/s42004-021-00578-z (PMC9814573; doi:10.1038/s42004-021-00578-z)
Supplement: Supplementary file 11 — Description of Additional Supplementary Files [file 42004_2021_578_MOESM11_ESM.pdf]

Description of Additional Supplementary Files

File Name: Supplementary Data 1

Description: The validation report for Sat1646-Mg^{2+} structure.

File Name: Supplementary Data 2

Description: The validation report for Sat1646 structure.

File Name: Supplementary Data 3

Description: The validation report for Stt4548 structure.

File Name: Supplementary Data 4

Description: The CIF file for structure of compound **3**.

File Name: Supplementary Data 5

Description: The CIF file for structure of compound **4**.

File Name: Supplementary Data 6

Description: The CIF file for structure of compound **5**.

File Name: Supplementary Data 7

Description: The CIF file for structure of compound **6**.
